# Selective Adsorption and Electrocatalysis of Polysulfides through Hexatomic Nickel Clusters Embedded in N-Doped Graphene toward High-Performance Li-S Batteries

**DOI:** 10.34133/2020/5714349

**Published:** 2020-06-26

**Authors:** Jiapeng Ji, Ying Sha, Zeheng Li, Xuehui Gao, Teng Zhang, Shiyu Zhou, Tong Qiu, Shaodong Zhou, Liang Zhang, Min Ling, Yanglong Hou, Chengdu Liang

**Affiliations:** ^1^Zhejiang Provincial Key Laboratory of Advanced Chemical Engineering Manufacture Technology, College of Chemical and Biological Engineering, Zhejiang University, Hangzhou 310027, China; ^2^Beijing Key Laboratory for Magnetoelectric Materials and Devices, Beijing Innovation Center for Engineering Science and Advanced Technology (BIC-ESAT), Department of Materials Science and Engineering, College of Engineering, Peking University, Beijing 100871, China; ^3^Institute of Functional Nano & Soft Materials (FUNSOM), Jiangsu Key Laboratory for Carbon-Based Functional Materials & Devices, Joint International Research Laboratory of Carbon-Based Functional Materials and Devices, Soochow University, Suzhou, 215123 Jiangsu, China

## Abstract

The shuttle effect hinders the practical application of lithium-sulfur (Li-S) batteries due to the poor affinity between a substrate and Li polysulfides (LiPSs) and the sluggish transition of soluble LiPSs to insoluble Li_2_S or elemental S. Here, we report that Ni hexatomic clusters embedded in a nitrogen-doped three-dimensional (3D) graphene framework (Ni-N/G) possess stronger interaction with soluble polysulfides than that with insoluble polysulfides. The synthetic electrocatalyst deployed in the sulfur cathode plays a multifunctional role: (i) selectively adsorbing the polysulfides dissolved in the electrolyte, (ii) expediting the sluggish liquid-solid phase transformations at the active sites as electrocatalysts, and (iii) accelerating the kinetics of the electrochemical reaction of multielectron sulfur, thereby inhibiting the dissolution of LiPSs. The constructed S@Ni-N/G cathode delivers an areal capacity of 9.43 mAh cm^−2^ at 0.1 C at S loading of 6.8 mg cm^−2^, and it exhibits a gravimetric capacity of 1104 mAh g^−1^ with a capacity fading rate of 0.045% per cycle over 50 cycles at 0.2 C at S loading of 2.0 mg cm^−2^. This work opens a rational approach to achieve the selective adsorption and expediting of polysulfide transition for the performance enhancement of Li-S batteries.

## 1. Introduction

In imminent pursuit of next-generation electrical energy storage (EES) technologies, lithium-sulfur (Li-S) batteries have attracted enormous research interests due to the high sulfur-specific capacity of 1675 mAh g^−1^ and the earth-abundant sulfur sources [1–4]. However, the practical application of Li-S batteries is hindered by multiple challenges, i.e., the insulation of S and its discharge products (Li_2_S_2_/Li_2_S), the large volume fluctuation, and the flagrant dissolution of Li_2_S*_x_* (4 ≤ *x* ≤ 8) into the electrolyte during charge/discharge cycles [5]. One common countermeasure to address the shuttling effects is to adsorb Li polysulfides (LiPSs) through the porous carbon hosts [6], binder [7], and membrane [8, 9]. Another recently emerging alternative is the use of electrocatalysts in the cathode to accelerate the conversion of soluble LiPSs to insoluble end products (sulfur in the charge reaction and Li_2_S in the discharge reaction), thereby reducing the polysulfide presence in the electrolyte [10–12]. Similar to adsorbent materials, an excellent electrocatalyst in the sulfur cathode must also have strong interaction with LiPSs, together with good electronic conductivity and electrochemical stability simultaneously.

Nonetheless, most adsorbent materials or electrocatalysts reported to date have not paid close attention to selective adsorption of specific polysulfide. Specifically, adsorption sites have strong interaction with both soluble polysulfide (Li_2_S*_x_*, 4 ≤ *x* ≤ 8) and insoluble polysulfide (Li_2_S_2_/Li_2_S), which cannot preferentially adsorb the former [13–17]. Moreover, the entropy-reduced liquid-solid phase transformations of soluble LiPSs and insoluble LiPSs at the catalytic sites have to overcome certain energy barriers [12]. As a result, active sites are mainly occupied by insoluble polysulfide with stronger interaction and the catalytic activity gradually declines. We propose here to exploit an electrocatalyst, which not only has stronger interaction with soluble polysulfides than that with insoluble polysulfides but also can selectively collect the polysulfide dissolved in the electrolyte and constantly “kick off” the immobilized insoluble polysulfide at the catalytic site, thus accelerating the kinetics of the electrochemical reaction of multielectron sulfur and reducing the dissolution of Li_2_S*_x_* (4 ≤ *x* ≤ 8) into the electrolyte.

Here, in order to confirm our concept, we intentionally prepared an N-doped 3D graphene framework embedded with Ni atomic clusters (Ni-N/G) as a sulfur cathode host material for Li-S batteries. The morphology and coordination configuration of Ni clusters at the atomic level were identified by high-angle annular dark-field imaging-scanning transmission electron microscopy (HAADF-STEM) and synchrotron X-ray absorption spectroscopy. Density functional theory (DFT) calculation results further revealed that the atomic-scale Ni_6_-N-C configuration possesses distinctive binding energies of LiPS species, which effectively inhibit the dissolution and enhance the conversion of LiPSs. Along with high electrical conductivity, hierarchically porous 3D structure, and large specific surface, the synthetic electrocatalyst exhibits impressive electrochemical performances in Li-S batteries. This work illustrates the important role of selective adsorption in inhibiting dissolution and enhancing the redox kinetics of polysulfides.

## 2. Results and Discussion

### 2.1. Synthesis and Characterization

**Figure 1:**
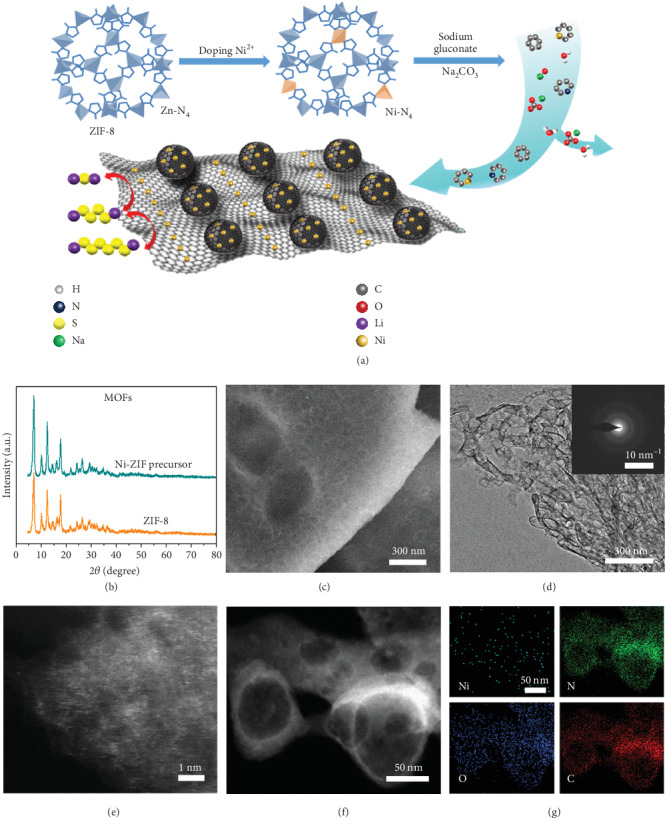
Synthesis and morphological characterization of the Ni-N/G. (a) Schematic illustration of the synthesis of the 3D graphene framework decorated with nickel atomic clusters (Ni-N/G). (b) XRD patterns of ZIF-8 precursors and Ni-doped ZIF-8 precursors. Representative electron microscopy images. (c) SEM, (d) TEM (inset, corresponding SAEDP), (e) HADDF-STEM, and (f) STEM images of the Ni-N/G sample and (g) corresponding EDX maps (colours) of an individual element.

The synthesis procedure of Ni-N/G is schematically illustrated in Figure 1(a) [18–20]. Firstly, active Ni species were introduced during the growth of a zinc-based zeolite imidazole framework (ZIF-8) by simultaneously adding Ni and Zn ions with 2-methylimidazole in methanol at 60°C. Tetrahedral Ni-N_4_ structures are likely existent in the precursors of Zn-N_4_ complexes, as there is no change in the identical XRD pattern obtained before and after doping (Figure 1(b)) [20]. Subsequently, the precursors were carbonized and reconstructed with sodium gluconate carbon (GS) in a nitrogen atmosphere. During the heating process, graphene nanosheets (GNs) were formed through thermal cyclodehydration and in-plane carbon reconstruction of sodium gluconate carbon in molten Na_2_CO_3_ media. Once the temperature exceeded 907°C, Zn in ZIFs was violently evaporated, resulting in porous carbon structures, and Ni-doped ZIFs were broken into random cyclized carbonaceous radicals modified with heteroatom Ni and N [19]. Finally, Ni species were anchored by nitrogen atoms and in situ atomically dispersed in a hierarchical graphene framework through carbothermal reduction.

The morphology and structures of samples were characterized by scanning electron microscopy (SEM) and X-ray diffraction (XRD) analysis. SEM images confirm that Ni-N/G has porous sphere framework morphology similar to the plant cell wall (Figure 1(c)), whereas the control samples without the introduction of Ni, gs, or Ni-ZIFs (N/G, Ni-N/C, and GNs) have entirely different morphologies (Figure S1). Transmission electron microscopy (TEM) images further demonstrate the 3D hierarchically porous framework consisting of abundant nanopores (~50 nm in diameter) interconnected with each other by graphene (Figure 1(d)). No Ni-derived nanoparticles are detected in high-resolution TEM (HRTEM) except for the lattice stripes of graphene (Figure S2). The N/G composite and GNs along with wrinkled surfaces also exhibit a characteristic graphene diffraction ring, consistent with the former research (Figures S3-S4) [21]. The size of independent carbonized Ni-ZIF particles (Ni-N/C) is around 250 nm (Figure S5). The structure of Ni-N/G at the atomic scale is further revealed by high-angle annular dark-field (HAADF) imaging with aberration-corrected scanning TEM (STEM). As shown in Figure 1(e), numerous individual bright spots can be seen, which are attributed to atomically dispersed Ni on the surface of Ni-N/G [22]. The distinct spatial distributions of Ni, C, N, and O elements for these four samples are confirmed by the energy-dispersive X-ray spectroscopy (EDX) elemental mapping images (Figures 1(f) and 1(g) and Figures S6-S9, Table S1), suggesting the uniform distribution of nickel and nitrogen elements.

**Figure 2:**
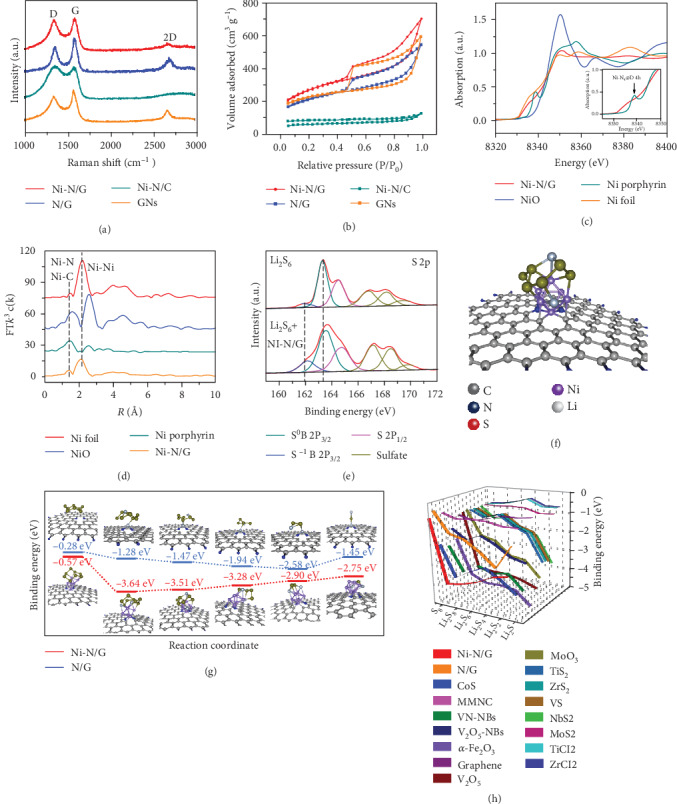
Structural characterizations and theoretical calculations. (a) Raman spectra and (b) N_2_ adsorption/desorption isotherms of Ni-N/G, N/G, Ni-N/C, and GNs. (c) Ni *K*-edge XANES spectra (inset, the enlarged spectra of Ni porphyrin and Ni-N/G) and (d) Ni *K*-edge *k*^3^-weighted FT-EXAFS spectra of Ni foil, NiO, and Ni porphyrin and Ni-N/G. Experimental testing and theoretical simulation of the polysulfide adsorption and conversion ability of Ni-N/G. (e) XPS S 2p spectra of Li_2_S_6_ and Li_2_S_6_/Ni-N/G. (f) Structure of Ni_6_-N/C used in first-principle calculations. (g) Binding geometric configurations and binding energies of sulfur species with the N/G and Ni-N/G. The high binding energies indicate the strong adsorption of Ni-N/G with sulfur species. (h) The binding energies between different lithium polysulfides and adsorbent materials, based on Ni-N/G compared with other currently reported sulfur host materials in the literature. Energy unit: electronvolts. Zero-point corrected.

XRD patterns confirm the formation of graphene carbon in Ni-N/G during pyrolysis. As shown in Figure S10, a broad peak centered at 25.3° is observed in Ni-N/G, corresponding to the characteristic peaks of graphene and implying that the stacking of graphene is not well ordered [19]. The same phenomena are observed in another two contrast samples, namely, N/G and GNs. However, when sodium gluconate was removed from the reaction system, i.e., Ni-N/C, the graphene peak is accompanied by the emergence of a new graphite peak (002) at 26.2°. The graphitic nature of Ni-N/G, N/G, Ni-N/C, and GNs were further corroborated by Raman spectra. As shown in Figure 2(a), the well-resolved D (1344 cm^−1^), G (1577 cm^−1^), and 2D (2689 cm^−1^) bands are observed in Ni-N/G, N/G, and GN samples, whereas blocky Ni-N/C exhibits no 2D band. These results are consistent with those of morphology characterization, indicating that the crisscrossed 3D graphene framework is derived from carbonization and reconstruction of GS. By comparing the *I*_D_/*I*_G_ values of Ni-N/G and N/G, it is inferred that Ni doping may induce more defects on the graphene surface [20]. The nitrogen adsorption-desorption isotherm and corresponding pore size distributions indicate that Ni-N/G possesses an ultrahigh Brunauer-Emmett-Teller (BET) surface area of 919.9 m^2^ g^−1^, a mesoporous size distribution centered at ~3.86 nm, and a total pore volume of 0.948 cm^3^ g^−1^ (Figures 2(b) and Figure S11, Table S2). Due to the huge specific surface area and pore volume, 71.44 to 75.26 wt% of molten S_8_ was filled into Ni-N/G and the control samples in a short time, which is confirmed by TGA curves in Figure S12.

The valence states of isolated C, N, and O atoms were investigated by X-ray photoelectron spectroscopy (XPS). As indicated in the C 1s and N 1s XPS spectra of Ni-N/G and N/G (Figures S13-S14), graphitic C dominates the C 1s spectrum and nitrogen atoms doped into the graphene framework are present in the forms of pyridinic (~398.8 eV), pyrrolic (~400.2 eV), graphitic (~401.5 eV), and oxidized (~403.3 eV) N species [11]. As shown in the N 1s XPS spectra and element content of Ni-N/C and GNs (Figures S15-S16 and Tables S3-S4), when N_2_ was the only nitrogen source, pyrrolic N and graphitic N dominate in GNs; after the introduction of dimethylimidazole, pyridinic nitrogen becomes the main nitrogen form in Ni-N/C. Thus, it can be drawn that pyridinic nitrogen was mainly derived from the preserved Ni-N structure in carbonizing Ni-doped ZIFs, while the pyrrolic nitrogen and graphitic nitrogen were mainly introduced by N_2_. Furthermore, Ni-N/G possesses a high content of graphitic carbon and graphitic nitrogen, introducing extra electrons into the delocalized system, which can significantly improve the conductivity [23].

To further investigate the chemical state and local coordination structure of Ni atoms in Ni-N/G, X-ray absorption near-edge structure (XANES) and extended X-ray fine structure (EXAFS) spectroscope measurements were employed (Figures 2(c) and 2(d) and Figure S17). The results for Ni-N/G are shown along with those for the reference sample Ni foil, NiO, and Ni porphyrin (Figure 2(c)). The XANES profile of Ni-N/G shows a significant difference from those of the reference samples. The preedge peak at 8338 eV in the XANES is a fingerprint featuring the Ni-N_4_ square planar D_4h_ symmetry in the reference Ni porphyrin (Figure 2(c), inset). But the corresponding preedge peak in Ni-N/G is rather weak due to the serious distortion of the Ni-N_4_ planar structure or replacement of integrant N atoms [24]. The *K*-edge WL intensities of Ni-N/G are between those of the reference samples Ni foil and NiO, indicating that Ni atoms in Ni-N/G are positively charged.

The coordination environment of Ni atoms in Ni-N/G was further elucidated by Fourier transformations (FT) of *k*^3^-weighted EXAFS (FT-EXAFS) (Figure 2(d)) [24, 25]. The oscillation of Ni-N/G is similar to that of bulk Ni, confirming that Ni clusters are in similar atomic local structure configuration of bulk Ni. But the amplitude of the Ni sample is expected to attenuate significantly with respect to the reference bulk sample, indicating the reduced size and decrease in the average coordination number [26]. Meanwhile, compared to the FT-EXAFS of the reference Ni porphyrin, which shows a symmetric FT peak (Ni-N_4_), Ni-N/G has a well-separated FT peak featuring shorter and longer bond distances of 1.42 Å and 2.08 Å, which correspond to Ni-N/C and Ni-Ni, respectively. With the EXAFS fitting process in *R* and *k* spaces, the coordination numbers of Ni-Ni, Ni-N, and Ni-C in Ni-N/G are calculated to be 5.7 (±0.41), 0.33 (±0.05), and 0.54 (±0.05), respectively (Figure S17 and Table S5). Combining XAS analyses with the observation of TEM and HADDF-STEM, it is confirmed that Ni clusters are atomically embedded in the N-doped 3D graphene framework and are coordinated by N atoms and C atoms to form Ni_6_-N-C coordination centers.

### 2.2. Adsorption Experiments and Theoretical Calculations

To experimentally evaluate the adsorption ability of cell wall-like frameworks with LiPSs, a visualized adsorption test was conducted by adding Ni-N/G, N/G, Ni-N/C, and GNs into a solution of 0.005 M Li_2_S_6_ in tetrahydrofuran (THF). And the Li_2_S_6_/THF solution filled in a blank vial without any addition was regarded as a comparison. As shown in Figure S18, the Ni-N/G sample with Ni clusters has almost decoloured the LiPS solution after the mixture was settled down for 4 h, which is much more obvious than other control samples. The interaction between Ni-N/G and Li_2_S_6_ was investigated by X-ray photoelectron spectroscopy (XPS). The spectrum of Li_2_S_6_ (Figure 2(e)) shows the S 2p_3/2_ at 161.9 and 163.3 eV, representing the terminal sulfur (S^−1^T) and bridging sulfur (S^0^B), respectively [27]. The spectrum of Li_2_S_6_+Ni-N/G exhibits two higher binding energy contributions at 162.2 eV and 163.6 eV (Figure 2(e)), representing a shift of +0.3 eV for both S^−1^T and S^0^B, respectively. This can be explained by the fact that the polysulfide provides a large amount of electron density to the Ni; thus, S has higher binding energy. Additionally, the significant enhancement of binding energy at 162.2 eV is attributed to the interaction of elemental sulfur with Ni-N/G (forming Ni-S). These results provide strong evidence for the chemical bonding of the terminal and bridging sulfur between LiPSs and the Ni-N/G.

To understand the mechanism of superior reaction kinetics of charge/discharge of the S@Ni-N/G cathode, density functional theory (DFT) simulation was conducted, where the binding energies of different LiPSs on Ni-N/G were investigated with N/G used as a reference [28, 29]. As shown in Figure S19, two optimized models of N-doped GNs with and without Ni clusters were considered in our simulation. Experimental results from XPS and XAS have shown that the Ni cluster is composed of six Ni atoms with the size of ~0.1 nm and bonded to pyridinic N atoms and carbon atoms. The optimized structures of the intermediates trapped by Ni-N/G and N/G substrates are identified, respectively, as displayed in Figures 2(f) and 2(g), and the binding energies between the Ni-N/G with S (-0.57 eV), Li_2_S_8_ (-3.64 eV), Li_2_S_6_ (-3.51 eV), Li_2_S_4_ (-3.28 eV), Li_2_S_2_ (-2.90 eV), and Li_2_S (-2.75 eV) species are calculated to be much higher than those of N/G (Table S6). These results suggest the strong interactions between the Ni-N/G composite and sulfur species, well consistent with the experimental adsorption.

During the discharging process, the binding energy of Li_2_S_8_-Ni_6_-N-C (-3.64 eV) is much more negative than that of S_8_-Ni_6_-N-C (-0.57 eV), indicating that the spontaneous exothermic conversion reaction from S_8_ into Li_2_S_8_ is kinetically fast [11]. In the subsequent steps to form Li_2_S_6_, Li_2_S_4_, Li_2_S_2_, and Li_2_S, the binding energy of the reactants is always greater than that of the products, which helps kick off the products from the catalytic Ni_6_ sites and thus speed up the conversion of LiPSs, especially the immobilized Li_2_S_2_/Li_2_S. In the charging process, the immobilized S at the active site can also be easily replaced by polysulfides, due to the huge binding energy gap. The binding energies between different lithium polysulfides and substrates based on Ni-N/G are compared with those from other currently reported sulfur host materials (Figure 2(h) and Table S6) [13–17]. For the phase transition process (Equations (1) and (2)), it is clearly a process of entropy reduction. According to the Gibbs free energy (Equation (3)), only when *∆H* ≪ 0 can the process happen spontaneously. Unfortunately, few sulfur host materials in the literature meet this requirement, whereas the binding energy of Li_2_S_8_ on Ni-N/G is much stronger than that of Li_2_S_2_/Li_2_S, reducing the energy barriers in liquid-solid phase transformations. Therefore, the integrated Ni_6_-N-C configuration in the Ni-N/G composite has multifunctions: selectively adsorbing the soluble polysulfide, expediting the liquid-solid phase transformations, and accelerating the kinetics of polysulfide redox, demonstrating the application potential in enhancing the performance of Li-S batteries.

(1) $$\begin{array}{c} {\text{Li}}_{2}{\text{S}}_{8\left(\text{l}\right)}+\text{L}{\text{i}}_{2}\text{S}@{\text{Site}}_{\left(\text{s}\right)}⟶{\text{Li}}_{2}{\text{S}}_{8}@{\text{Site}}_{\left(\text{s}\right)}+\text{L}{\text{i}}_{2}{\text{S}}_{\left(\text{s}\right)} \end{array}$$

(2) $$\begin{array}{c} \Delta H=E_{\text{b}}\left({\text{Li}}_{2}{\text{S}}_{8}@{\text{Site}}_{\left(\text{s}\right)}\right)-E_{\text{b}}\left({\text{Li}}_{2}\text{S}@{\text{Site}}_{\left(\text{s}\right)}\right), \end{array}$$

(3) $$\begin{array}{c} \Delta G=\Delta H-T\Delta S. \end{array}$$

### 2.3. Electrochemical Properties

**Figure 3:**
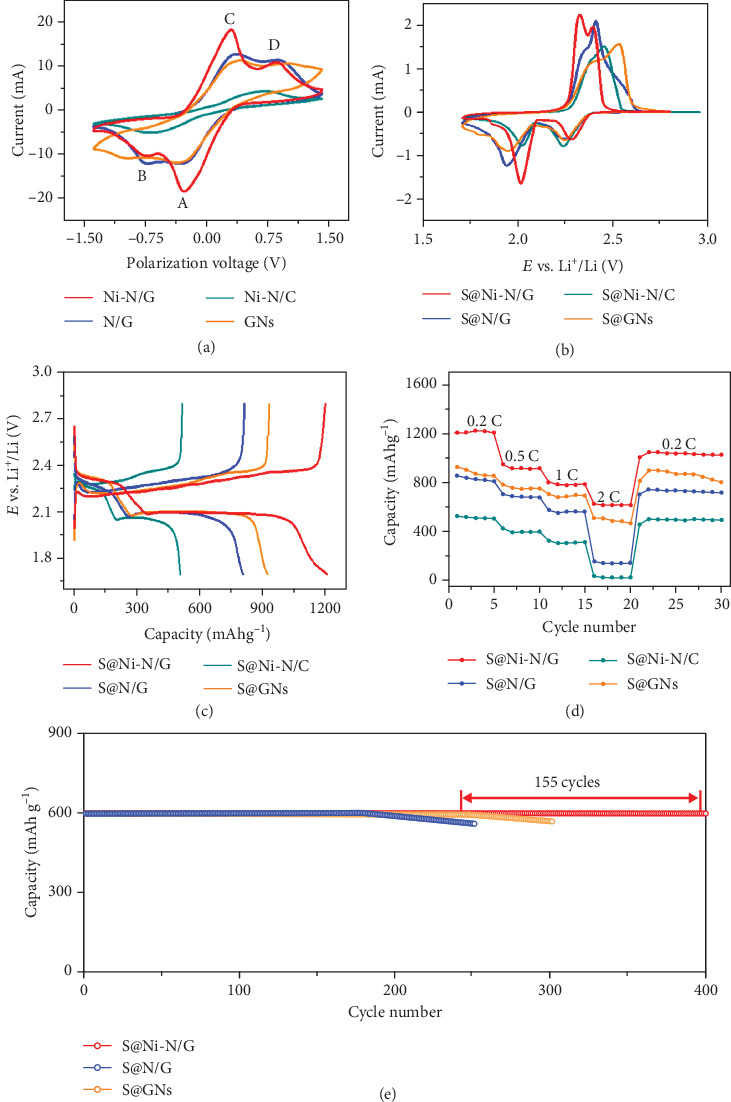
Electrochemical performances of S@Ni-N/G, S@N/G, S@Ni-N/C, and S@GN cathodes in Li-S batteries with the sulfur loading of 1.0 mg cm^−2^. (a) CV curves of symmetric cells over a voltage between -1.4 and 1.4 V with a sweep rate of 10 mV s^−1^. (b) CV curves over a voltage range of 1.7-2.8 V with a sweep rate of 0.2 mV s^−1^. (c) Discharge-charge profiles, (d) rate capabilities, and (e) cycle performance with limited discharge capacity of 600 mAh g^−1^ at 430 mA g^−1^.

To reveal the electrochemical conversion kinetics of LiPSs on Ni-N/G, N/G, Ni-N/C, and GN electrodes, cyclic voltammetry (CV) of symmetric cells was conducted in 0.5 M Li2S6 electrolyte, with a voltage window of -1.4 and 1.4 V and a scan rate of 10 mV s-1, using identical working and counter electrodes [11]. As shown in Figure 3(a), the CV curve of the Ni-N/G electrode exhibits four distinct redox peaks, which can be assigned to the electrochemical reactions of LiPSs on the electrodes. The peaks in the negative scan can be assigned to the reduction of S to soluble Li_2_S_6_/Li_2_S_4_ (peak A, -0.29 V) and the further reduction into insoluble Li_2_S_2_/Li_2_S (peak B, -0.75 V) on the working electrode, whereas the peaks in the positive scan are accompanied by the decomposition of Li_2_S_2_/Li_2_S, the reconstitution of Li_2_S_6_/Li_2_S_4_ (peak C, 0.29 V), and further the oxidation to generate elemental S (peak D, 0.82 V) [11]. The CV curves of N/G, Ni-N/C, and GN electrodes also show four redox peaks, but the intensities are much lower, and the voltage differences between the cathodic and anodic peaks are higher than those of Ni-N/G. Thus, these CV curves conducted on symmetric cells demonstrate that the Ni hexatomic cluster plays a critical role in improving electrochemical kinetics of LiPS conversion.

The electrochemical properties of S@Ni-N/G and the control samples as sulfur cathodes were evaluated systematically through assembling Li-S coin cells.  Figure 3(b) demonstrates the cyclic voltammetry (CV) curves of S@Ni-N/G, S@N/G, S@Ni-N/C, and S@GN cathodes with the sulfur loading of 1.0 mg cm^−2^ at a scanning rate of 0.2 mV s^−1^ [30]. It can be identified that there are two cathodic peaks and one anodic peak in the CV curve of the S@Ni-N/G cell. The cathodic peak at 2.28 V is attributed to the formation of soluble polysulfides (Li_2_S*_x_*, 4 < *x* ≤ 8) from sulfur, and the peak at 2.02 V is ascribed to the formation of insoluble sulfides (Li_2_S_2_ and Li_2_S), respectively. The single anodic peak at 2.32 V is due to the one-step oxidation of Li_2_S/Li_2_S_2_ into S_8_ [14]. In comparison, the CV curves of the cells with the control samples exhibit an obvious negative shift for the two reduction peaks, a positive shift for the single oxidation peak, and lower current density. To further confirm the electrocatalytic effect of Ni-N/G on the electrochemical redox conversion of LiPSs, the onset potentials were taken at a current density of 10 *μ*A cm^−2^ according to previous reports (Figure S20) [13, 15]. Compared to S@N/G, S@Ni-N/C, and S@GN control samples, the introduction of Ni clusters increases the onset potentials of cathodic peaks and decreases that of the anodic peak. Since the Ni-N/G and N/G cells have identical test conditions, their overpotential difference is mainly attributed to the existence of Ni_6_ catalytic sites. These results demonstrate that the Ni-N/G structure can significantly improve the utilization of sulfur species, accelerate electrocatalytic effect, and enhance LiPS redox kinetics. In addition, four CV curve cycles were performed to investigate the reversibility of the S@Ni-N/G cathode in Figure S21. Compared with the initial CV cycle, the following ones remain almost unchanged, implying the good cycling stability of the S@Ni-N/G cathode.

Figure 3(c) shows the galvanostatic charge/discharge profiles of the four electrodes with S mass loading of 1.0 mg cm^−2^. The S@Ni-N/G electrode possesses a considerably high initial discharge capacity of 1204 mAh g^−1^ at 0.2 C. When the current rate increases to 0.5, 1, and 2 C, the discharge capacities of the S@Ni-N/G cathode achieve 967, 822, and 625 mAh g^−1^, respectively (Figures 3(d) and Figure S22). Conversely, the discharge capacities of the control samples are seriously attenuated under the same condition, indicating the modified rate performance of S@Ni-N/G.  Figure 3(e) displays the cycle performance of S@Ni-N/G, S@N/G, and S@GN cathodes with a charge/discharge capacity limited to 600 mAh g^−1^ at 430 mA g^−1^. It shows that the S@Ni-N/G anode with Ni clusters can hold the capacity of 600 mAh g^−1^ for 400 cycles, whereas the S@N/G and S@GN cathodes perform 190 and 245 cycles with the capacity of 600 mAh g^−1^. The significantly longer cycle life of the S@Ni-N/G indicates that the integrated Ni_6_-N-C configuration in the Ni-N/G composite provides stronger chemisorption of LiPSs, which ensures a great capability to restrict polysulfide dissolution and thus a superior cycling performance.

At a higher rate of 1.0 C, the S@Ni-N/G cathode still delivers the highest initial discharge capacity of 854.9 mAh g^−1^ and maintains at 537.5 mAh g^−1^ after 400 cycles (Figure S23). The resistance characteristics of these cathodes were investigated by electrochemical impedance spectroscopy (EIS) (Figure S24 and Tables S7-S8). Benefitting from the high content of graphitic carbon and graphitic nitrogen in the materials, these cathodes exhibit small charge transfer resistance. After 400 cycles, the diffusion resistance of the control sample is significantly enlarged or the display frequency regions become limited, indicating that the chemical system dynamics become sluggish [31]. The S@Ni-N/G cathode maintains very low charge transfer resistance, indicating that the N doping and the catalytic effect of Ni monoatoms can facilitate the electron transportation and accelerate the kinetics of polysulfide conversion [11]. In order to illustrate the contribution of Ni-N/G to the total discharge capacity of Li-S batteries, Ni-N/G (without the filling of sulfur) was tested as the cathode material for LIBs within a voltage window of 1.7-2.8 V vs. Li/Li^+^ (Figure S25). The mingy specific capacities indicate that the pristine Ni-N/G has almost no lithium storage capacity.

**Figure 4:**
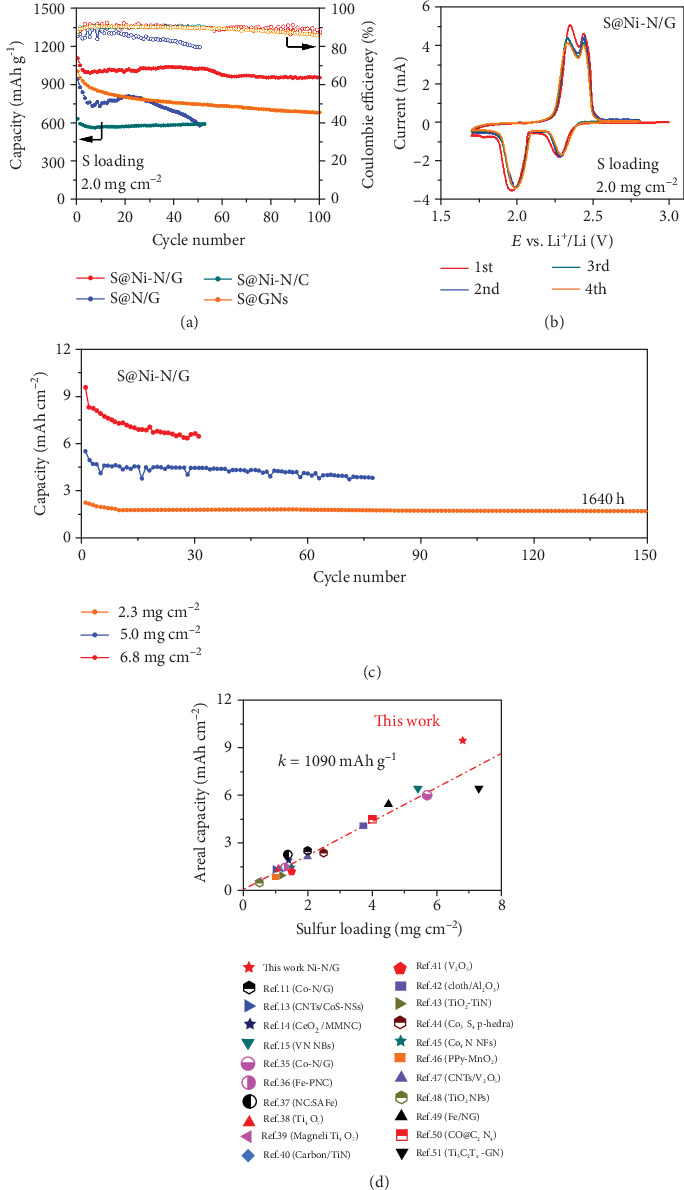
Electrochemical performances with high areal loading of sulfur. (a) Cycling performances and Coulombic efficiencies of S@Ni-N/G and control cathodes under 0.2 C. (b) CV curves over a voltage range of 1.7-2.8 V with sweep rate of 0.2 mV s^−1^. (c) Cycling performance of S@Ni-N/G with different areal loading of sulfur at 0.1 C. (d) Areal capacity of the S@Ni-N/G cathode with sulfur loading of 6.8 mg cm^−2^ at 0.1 C compared with that of other currently reported sulfur host materials with catalytic properties in the literature.

According to the U.S. Department of Energy (DOE), lithium-ion batteries must meet the following requirements for large-scale EES: shorter discharge times (from seconds to 6 hours), high energy densities, high efficiency (60-95%), and low self-discharge compared to other storage technology types [32–34]. In consideration of these factors, cathodes with higher areal sulfur loading weights are called for.  Figure 4(a) presents the cycling performances of S@Ni-N/G and control samples with the sulfur loading of 2.0 mg cm^−2^ under 0.2 C. The S@Ni-N/G cathode delivers an initial discharge capacity of 1103.6 mAh g^−1^, and it maintains a stable cycling performance (953.5 mAh g^−1^) and high Coulombic efficiency of 97% after 100 cycles. In contrast, the discharge capacities and Coulombic efficiency of the other three control cathodes decrease significantly during the cycles. The CV curves of the S@Ni-N/G cathode exhibit high peak current intensity (~3.4 mA) and small voltage hysteresis between redox peaks (Figure 4(b)). Moreover, the excellent reversibility of CV curves indicates the good cycling stability of the S@Ni-N/G thick cathode.

To explore the practical application of S@Ni-N/G, the S mass loading was further increased to 2.3, 5.0, and 6.8 mg cm^−2^ in cathodes at 0.1 C, corresponding to 0.39, 0.84, and 1.14 mA cm^−2^, respectively (Figures 4(c) and Figure S26). The composites delivered initial areal specific capacities of 2.22, 5.52, and 9.43 mAh cm^−2^. Most interestingly, it is found statistically that the positions of the reported sulfur cathode materials are distributed in a straight line with a slope of 1090 mAh g^−1^, and the Ni-N/G composite is located above the straightness, indicating its superior performance (Figure 4(d) and Table S9) [11, 13–15, 35–51]. Moreover, the S@Ni-N/G cathode with high sulfur loading of 2.3 mg cm^−2^ under 0.1 C shows good cycling performances and deliver the discharge capacities of 734 mAh g^−1^ after cycling for 150 cycles, namely, 1460 h, and the corresponding capacity retentions are 76.1% compared to the initial cycle. The outstanding performances of S@Ni-N/G cathodes with high sulfur loading are ascribed to the strong polysulfide adsorption/confinement, high specific surface, and high conductivity provided by codoped Ni-N atoms and the 3D graphene framework, which facilitate the electron/ion transport and ensure the efficient sulfur utilization in the thick electrodes.

On the basis of the excellent charge/discharge performance of Ni-N/G composite at 0.1 C, a soft-packed Li-S battery of the S@Ni-N/G cathode was assembled with a correspondingly shaped Celgard membrane and lithium foil as the separator and anode, respectively. The sandwich structure was sealed by Al plastic films straight after injecting electrolyte, with the total weight of 2.37 g (Figure S27). The cycling performance test of the S@Ni-N/G soft-packed battery was divided into two phases: first five cycles at 1.94 mA and then the following cycles at 6.45 mA. The initial discharge capacity reached 51.4 mAh (6.1 mAh cm^−2^) and thereafter tended to be stable to 20.7 mAh (2.5 mAh cm^−2^). The corresponding average Coulombic efficiency was up to 96%, suggesting the fine suppression of the polysulfide shuttle effect and the potential of the Ni-N/G composite in the practical application of EES.

## 3. Conclusion

In summary, Ni hexatomic clusters embedded in the N-doped 3D graphene framework were prepared as both polysulfide adsorbents and electrocatalysts in rechargeable Li-S batteries. Ni-N/G displays significant selectivity differences in the adsorption of soluble and insoluble polysulfides. The selective adsorption and catalytic properties were proven by experimental measurements and density functional theory (DFT) calculations. When deployed in the sulfur cathode, the S@Ni-N/G cathode possesses the following merits: (i) atomically dispersed Ni derived from metal doped ZIF-8 effectively acts as an anchoring conversion center of LiPSs, (ii) the crisscrossed 3D hierarchically graphene framework and nitrogen doping ensure good stability and small charge transfer resistance, and (iii) the porous nanostructure enables high sulfur loading and accommodates the volume change of sulfur. This work figures out one approach to design an electrocatalyst to selectively adsorb and convert polysulfides for high performance of Li-S batteries.

## 4. Materials and Methods

### 4.1. Reagents

Anhydrous sodium carbonate (Na_2_CO_3_), hydrochloric acid (HCl), nickel nitrate hexahydrate (Ni(NO_3_)_2_·6H_2_O), zinc nitrate hexahydrate (Zn(NO_3_)_2_·6H_2_O), and absolute methanol (CH_3_OH) were obtained from Shanghai Chemical Reagents, China. Sublimed sulfur, N-methyl pyrrolidone (NMP), and 2-methylimidazol (C_4_H_6_N_2_, 98%) were purchased from Aladdin. Sodium gluconate was acquired from 3A Chemicals of Shanghai, China. Polyvinylidene fluoride (PVDF) and Super P Li were obtained from Taiyuan Yingze District Lizhiyuan Battery Sales Department. Lithium sulfur electrolyte (LS009) containing 1.0 M bis(trifluoromethane) sulfonamide lithium salt (LiTFSI) in a mixed solvent of dimethoxyethane and 1,3-dioxolane (DME/DOL, 1 : 1 volume ratio) with 2 wt% of lithium nitrate was purchased from the DoDoChem. The chemicals of this experiment were of analytical grade without further purification.

### 4.2. Synthesis of Ni-ZIF and ZIF

Ni-doped ZIF precursors were synthesized at 60°C in a methanol solution. Ni(NO_3_)_2_·6H_2_O (72 mg) and Zn(NO_3_)_2_·6H_2_O (3.39 g) were dissolved in 600 mL methanol (marked A). 2-Methylimidazole (3.94 g) was dissolved in 600 mL methanol (marked B). Next, A was poured into B and sealed. The mixed solution was heated to 60°C and maintained for 24 h. Then, centrifugation was performed to collect the precipitant with thorough ethanol washing, and the precipitant was dried at 60°C in a vacuum oven for 12 h. The only difference between the synthesis of ZIF and Ni-ZIF was that ZIF synthesis did not use Ni(NO_3_)_2_·H_2_O.

### 4.3. Synthesis of Ni-N/G, N/G, Ni-N/C, and GNs

Ni-N/G was synthesized by a molten salt method. Ni-doped ZIF (1 g), Na_2_CO_3_ (10 g), and sodium gluconate (5 g) were mixed together and ground in uniformity. Subsequently, the mixed precipitant was heated in a tube furnace under N_2_ flow from room temperature to 950°C with 3°C min^−1^ and kept at 950°C for 10 min. The acquired black mixture needed to react with 3 mol L^−1^ HCl solution until no bubbles appeared, and then, using DI water, the Ni-N/G was washed adequately to neutral. Finally, the precipitant was dried at 60°C for 12 h. The synthesis method of N/G was the same as Ni-N/G synthesis. The only difference was the replacement of Ni-doped ZIF (1 g) with ZIF (1 g). The synthesis method of Ni-N/C was the same as Ni-N/G synthesis. The only difference was that Ni-N/C synthesis did not use Na_2_CO_3_ (10 g) and sodium gluconate (5 g). The synthesis method of GNs was the same as Ni-N/G synthesis. The only difference was that the pristine ZIF-8 (1 g) precursor was mixed with Na_2_CO_3_ (10 g) and sodium gluconate (5 g).

### 4.4. Polysulfide Static Adsorption Test and XPS Sample Preparation

Li_2_S_6_ was prepared by dissolving sublimed sulfur and lithium sulfide in tetrahydrofuran (THF) at a molar ratio of 5 : 1 under stirring. Then, the Li_2_S_6_/THF solution was dried at 35°C to acquire yellow powder Li_2_S_6_. All procedures were performed in an Ar-filled glovebox. The static adsorption test was performed by adding Ni-N/G, N/G, Ni-N/C, and GNs with the same weight of 10 mg into the sealed vials containing Li_2_S_6_ (0.005 M) in 10 mL tetrahydrofuran (THF), respectively. Then, the mixtures were settled down for 4 h after shaking to observe the colour variation. The Li_2_S_6_/THF solution, as a comparison, was filled in a blank vial without any addition. The XPS samples of Ni-N/G were acquired by heating the Li_2_S_6_/THF solution containing Ni-N/G at 35°C. All procedures were performed in an Ar-filled glovebox.

### 4.5. Material Characterization

X-ray diffractions (XRD) were recorded through an X'Pert PRO (PANalytical, Netherlands) instrument with Cu K radiation over the 2*q* range from 10° to 80°. Raman spectroscopy was performed using a Laser Confocal Raman Microspectroscopy (LabRAM HR Evolution, HORIBA Jobin Yvon). X-ray photoelectron spectroscopy (XPS) analyses were conducted with an Escalab 250Xi XPS system with an Al K*α* (1486.6 eV) source. Scanning electron microscopy (SEM) was performed on a Hitachi S-4800 field emission scanning electron microscope (FE-SEM) (5 kV). Transmission electron microscopy (TEM) and high-resolution transmission electron microscopy (HRTEM) were conducted with a JEM-2100F field emission TEM. Energy-dispersive X-ray (EDX) was obtained using JEOL-2200FS and double Cs-corrected STEM at the acceleration voltage of 200 kV. HAADF-STEM images were acquired on a FEI TITAN Chemi STEM equipped with a CEOS (Heidelberg, Germany) probe corrector, operating at 200 kV. The specific surface area and pore size distribution were measured by using nitrogen adsorption/desorption isotherms through the Brunauer-Emmett-Teller (BET, AUTOSORB-IQ2-MP) method at 77 K. The BJH method is selected to calculate the pore size distribution. Thermogravimetric analysis (TGA) was performed on a thermal analyzer (Pyris 1 TGA) in the temperature range from 50°C to 600°C under nitrogen flow. The X-ray absorption fine structure (XAFS) at the Ni K (*E*_0_ = 8333.0 eV) edge was performed at the BL14W1 beamline of Shanghai Synchrotron Radiation Facility (SSRF) operated at 3.5 GeV under the “top-up” mode with a constant current of 260 mA. The XAFS data were recorded under a transmission mode with ion chambers. The energy was calibrated accordingly to the absorption edge of pure Ni foil. Athena and Artemis codes were used to extract the data and fit the profiles. For the X-ray absorption near-edge structure (XANES) part, the experimental absorption coefficients as function of energies *μ*(*E*) were processed by background subtraction and normalization procedures and reported as “normalized absorption” with *E*_0_ = 8333.0 eV for the Ni-N/G sample, Ni foil, NiO, and Ni porphyrin standard. For the extended X-ray absorption fine structure (EXAFS) part, the Fourier transformed (FT) data in the *R* space were analyzed for Ni-Ni, Ni-N, and Ni-C contributions. The passive electron factors, S_0_^2^, were determined by fitting the experimental data on Ni foils and fixing the coordination number (CN) of Ni-Ni to be 12 and then fixed for further analysis of the measured samples. The parameters describing the electronic properties (e.g., correction to the photoelectron energy origin, *E*_0_) and local structure environment including CN, bond distance (*R*), and Debye-Waller factor around the absorbing atoms were allowed to vary during the fitting process. The fitted ranges for *k* and *R* spaces were selected to be *k* = 2-9 Å^−1^ with *R* = 1-3 Å (*k*^3^ weighted). We set the initial amplitude attenuation factor S_0_^2^ to be 0.85 and the Debye-Waller factor *σ*^2^ to be 0.003 Å^2^ for all the analyzed Ni-Ni, Ni-N, and Ni-C shells [25].

### 4.6. Electrochemical Tests

The sulfur was mixed with Ni-N/G and ground in uniformity. Then, the sulfur containing the Ni-N/G substrate was heated at 155°C for 4 h under an argon atmosphere. Other samples underwent the same procedure as Ni-N/G. Symmetric cells were assembled with two electrodes that consisted of a mixture of 80 wt% of the active material (Ni-N/G, N/G, Ni-N/C, or GNs) and 20 wt% of polyvinylidene fluoride (PVDF). The active material mass loading was 0.8–1.0 mg cm^−2^. The electrolyte was a dioxolane (DOL)/dimethoxyethane (DME) (volume ratio 1 : 1) mixture containing 1 M LiTFSI, 0.5 M Li_2_S_6_, and 2.0 wt% LiNO_3_. Cyclic voltammetry (CV) was performed between −1.4 and 1.4 V at the scan rate of 10 mV s^−1^. The Li-S batteries were assembled with both CR2025-type coin cells and soft-package batteries in an Ar-filled glovebox. To fabricate the working electrodes of Li-S batteries, a slurry was made by mixing the composite of S-loading Ni-N/G, conductive carbon additive (Super P), and a polymeric binder (PVDF) in a weight ratio of 7 : 2 : 1 using NMP as the solvent. The prepared slurry was coated on the Al foil and dried at 60°C in the vacuum oven for 8 h. Next, the electrode was cut into the shape of a coin by a punching machine. The average mass loading of the active materials on a pole piece was controlled in the range of 1.0 mg cm^−2^ to 6.8 mg cm^−2^. When the mass loading of the active materials is greater than 3 mg cm^−2^, the prepared slurry is coated with nickel foam. Both reference and counter electrodes used lithium metal. And the polymeric porous membrane (polypropylene) was used as the separator. The electrolyte is critical to the performance of Li-S batteries. When the electrolyte is insufficient, the electrode and separator are not fully wetted, the active material utilization rate is low, and the ion conductivity is poor. However, the excessive electrolyte reduces the mass energy density of the battery. Moreover, the electrolyte and sulfur react to form soluble LiPS, which reduces the battery performance. Therefore, to maximize energy density, the amount of electrolyte used must be considered based on the sulfur content. Taking the above issues into consideration, this work chooses different amounts of LS009 electrolyte (20 and 10 *μ*L mg^−1^) for low- and high-sulfur content. The galvanostatic charge-discharge measurements were performed in the LAND battery cycle test system with the potential range of 1.7 to 2.8 V (vs. Li/Li^+^) at different current densities in the range of 0.1 to 2 C rate (1 C rate∼1675 mA g^−1^). The current density and capacity of the electrode were calculated based on the weight of sulfur in the electrode. Cyclic voltammetry (CV) analysis was performed at a scan rate of 0.1 mV s^−1^ from 1.7 to 2.8 V (vs. Li/Li^+^) on a CHI660E electrochemical workstation (CH Instruments, China). Electrochemical impedance spectroscopy (EIS) was conducted in the frequency range from 0.01 Hz to 1000 kHz with an amplitude of 5 mV.

### 4.7. Theory Calculation

In this work, the geometry optimization and frequency analysis were performed using the extended tight-binding semiempirical program package (xTB) [28, 29]. The geometries were obtained with GFN0-xTB, while the energies were given by GFN2-xTB. All stationary points were optimized without symmetry constraint, and their nature was confirmed by vibrational frequency analysis. The adsorption energy was defined as *E*_(ad)_ = *E*_(ad/surf)_ − *E*_(surf)_ − *E*_(ad)_, where *E*_(ad/surf)_, *E*_(surf)_, and *E*_(ad)_ are the total energies of the adsorbates binding to the surface, clean surface, and free adsorbate in the gas phase, respectively.

## Supplementary Materials

Supplementary Materials Figure S1: SEM images of (a) N/G, (b) Ni-N/C, and (c) GNs. Figure S2: (a) TEM and (b) high-resolution TEM (HRTEM) of the Ni-N/G composite. Figure S3: representative electron microscopy images. (a) TEM (inset, corresponding SAEDP) and (b) HRTEM images of the N/G sample. Figure S4: representative electron microscopy images. (a–b) TEM (inset (a), corresponding SAEDP) and (c) HRTEM images of the GN sample. Figure S5: representative electron microscopy images. (a) TEM (inset, corresponding SAEDP) and (b) HRTEM images of the Ni-N/C sample. Figure S6: (a) HAADF-STEM image of the Ni-N/G sample and (b) corresponding EDX maps (colours) of an individual element. Figure S7: (a) STEM image of the N/G sample and (b–d) corresponding EDX maps (colours) of an individual element. Figure S8: (a) STEM image of the Ni-N/C sample and (b) corresponding EDX maps (colours) of an individual element. Figure S9: (a) STEM image of the GN sample and (b–d) corresponding EDX maps (colours) of an individual element. Figure S10: XRD patterns of Ni-N/G, N/G, Ni-N/C, and GNs. Figure S11: pore size distribution curves of Ni-N/G, N/G, Ni-N/C, and GNs. Figure S12: TGA curve of S@Ni-N/G, S@N/G, S@Ni-N/C, and S@GNs in an N_2_ atmosphere heated from room temperature to 600°C with the ramping rate of 10°C min^−1^. Figure S13: XPS high-resolution spectra of (a) C 1s, (b) N 1s, and (c) O 1s in Ni-N/G. Figure S14: XPS high-resolution spectra of (a) C 1s, (b) N 1s, and (c) O 1s in N/G. Figure S15: XPS high-resolution spectra of (a) C 1s, (b) N 1s, and (c) O 1s in Ni-N/C. Figure S16: XPS high-resolution spectra of (a) C 1s, (b) N 1s, and (c) O 1s in GNs. Figure S17: the EXAFS fitting in the *k* space (a) and *R* space (b) about the measured (green line) and fitting curves (red line) for Ni-N/G. Figure S18: experimental testing of the polysulfide adsorption of Ni-N/G, N/G, Ni-N/C, and GNs with Li2S6 as a representative lithium polysulfide. Figure S19: structure of N/G (a) and Ni-N/G (b) used in first-principle calculations. Figure S20: differential CV curves (a–d) of S@Ni-N/G, S@N/G, S@Ni-N/C, and S@GN cathodes verified from the CV profiles in Figure 3(b). The corresponding onset potentials of re5dox peaks are attained at the current density of 10 *μ*A cm^−2^. The baseline potentials and baseline current densities are defined as the values before the redox peaks, where the variation on current density is the smallest, namely, dI/dV = 0. Figure S21: electrochemical performances (a–d) of S@Ni-N/G, S@N/G, S@Ni-N/C, and S@GN cathodes in Li-S batteries with the sulfur loading of 1.0 mg cm^−2^. CV curves over a voltage range of 1.7-2.8 V with a sweep rate of 0.2 mV s^−1^. Figure S22: typical discharge-charge profiles of the S@Ni-N/G composite at different current densities. Figure S23: electrochemical performances of S@Ni-N/G, S@N/G, S@Ni-N/C, and S@GN cathodes in Li-S batteries with the sulfur loading of 1.0 mg cm^−2^. Long-term cycling performances and corresponding Coulombic efficiencies under 1.0 C. Figure S24: EIS spectra of S@Ni-N/G, S@N/G, S@Ni-N/C, and S@GN cathodes (a) before and (b) after long-term cycling, revealing the much smaller charge transfer resistance of the S@Ni-N/G cathode. Figure S25: cycling performance of Ni-N/G as the cathode material for LIBs within a voltage window of 1.7-2.8 V vs. Li/Li+. The ultralow specific capacities indicate that the lithium storage capacity of pristine Ni-N/G has no contribution to the total discharge capacity of Li-S batteries. Figure S26: cycling performance and Coulombic efficiencies of S@Ni-N/G with different areal loading of sulfur at 0.1 C. Figure S27: cycling performance and Coulombic efficiencies of the S@Ni-N/G-based soft-packed Li-S battery at 0.1 C. Inset: the photograph of the soft-packed Li-S battery assembled with the S@Ni-N/G cathode and lithium foil anode. Table S1: atomic percentage ratios of different elements in Ni-N/G according to the EDX results of HADDF-STEM. Table S2: BET surface area, pore volume, and pore diameter of Ni-N/G, N/G, Ni-N/C, and GNs. Table S3: atomic percentage ratios of different elements in Ni-N/G and control samples according to the results of XPS. Table S4: atomic percentage ratios of different N types in Ni-N/G and control samples according to the results of XPS. Table S5: EXAFS parameters for Ni-N/G, Ni foil, NiO, and Ni porphyrin. Table S6: the binding energies between different lithium polysulfides and substrates, based on Ni-N/G compared with other currently reported sulfur host materials in the literature. Energy unit: electronvolts. Zero-point corrected. Table S7: summary of electrochemical impedance spectroscopy of the initial Ni-N/G, N/G, Ni-N/C, and GN samples at open-circuit voltage. Table S8: summary of electrochemical impedance spectroscopy of the Ni-N/G, N/G, Ni-N/C, and GN samples at open-circuit voltage after 400 cycles at 1 C. Table S9: electrochemical performances of sulfur cathodes based on Ni-N/G compared with other currently reported sulfur host materials in the literature.
