# Supplementary material for: Selective Adsorption and Electrocatalysis of Polysulfides through Hexatomic Nickel Clusters Embedded in N-Doped Graphene toward High-Performance Li-S Batteries
Source: Research (Wash D C). 2020 Jun 26;2020:5714349. doi: 10.34133/2020/5714349 (PMC7335422; doi:10.34133/2020/5714349)
Supplement: Supplementary Materials — Figure S1: SEM images of (a) N/G, (b) Ni-N/C, and (c) GNs. Figure S2: (a) TEM and (b) high-resolution TEM (HRTEM) of the Ni-N/G composite. Figure S3: representative electron microscopy images. (a) TEM (inset, corresponding SAEDP) and (b) HRTEM images of the N/G sample. Figure S4: representative electron microscopy images. (a–b) TEM (inset (a), corresponding SAEDP) and (c) HRTEM images of the GN sample. Figure S5: representative electron microscopy images. (a) TEM (inset, corresponding SAEDP) and (b) HRTEM images of the Ni-N/C sample. Figure S6: (a) HAADF-STEM image of the Ni-N/G sample and (b) corresponding EDX maps (colours) of an individual element. Figure S7: (a) STEM image of the N/G sample and (b–d) corresponding EDX maps (colours) of an individual element. Figure S8: (a) STEM image of the Ni-N/C sample and (b) corresponding EDX maps (colours) of an individual element. Figure S9: (a) STEM image of the GN sample and (b–d) corresponding EDX maps (colours) of an individual element. Figure S10: XRD patterns of Ni-N/G, N/G, Ni-N/C, and GNs. Figure S11: pore size distribution curves of Ni-N/G, N/G, Ni-N/C, and GNs. Figure S12: TGA curve of S@Ni-N/G, S@N/G, S@Ni-N/C, and S@GNs in an N2 atmosphere heated from room temperature to 600°C with the ramping rate of 10°C min−1. Figure S13: XPS high-resolution spectra of (a) C 1s, (b) N 1s, and (c) O 1s in Ni-N/G. Figure S14: XPS high-resolution spectra of (a) C 1s, (b) N 1s, and (c) O 1s in N/G. Figure S15: XPS high-resolution spectra of (a) C 1s, (b) N 1s, and (c) O 1s in Ni-N/C. Figure S16: XPS high-resolution spectra of (a) C 1s, (b) N 1s, and (c) O 1s in GNs. Figure S17: the EXAFS fitting in the k space (a) and R space (b) about the measured (green line) and fitting curves (red line) for Ni-N/G. Figure S18: experimental testing of the polysulfide adsorption of Ni-N/G, N/G, Ni-N/C, and GNs with Li2S6 as a representative lithium polysulfide. Figure S19: structure of N/G (a) and Ni-N/G (b) used in first-principle calculation [file 5714349.f1.docx]

Supporting Information

Selective Adsorption and Electrocatalysis of Polysulfides through Hexatomic Nickel Clusters Embedded in N-Doped Graphene toward High-Performance Li-S Batteries

Jiapeng Ji,^1^ Ying Sha,^1^ Zeheng Li,^1^ Xuehui Gao,^1^ Teng Zhang,^2^ Shiyu Zhou,^1^ Tong Qiu,^1^ Shaodong Zhou,^1^ Liang Zhang,^3^ Min Ling,*^,1^ Yanglong Hou,*^,2^ Chengdu Liang^1^

^1^Zhejiang Provincial Key Laboratory of Advanced Chemical Engineering Manufacture Technology, College of Chemical and Biological Engineering, Zhejiang University, Hangzhou 310027, China

^2^Beijing Key Laboratory for Magnetoelectric Materials and Devices, Beijing Innovation Center for Engineering Science and Advanced Technology (BIC-ESAT), Department of Materials Science and Engineering College of Engineering, Peking University, Beijing 100871, China

^3^Institute of Functional Nano & Soft Materials (FUNSOM), Jiangsu Key Laboratory for Carbon-Based Functional Materials & Devices, Joint International Research Laboratory of Carbon-Based Functional Materials and Devices, Soochow University, Suzhou 215123, Jiangsu, China

Correspondence should be addressed to Min Ling; minling@zju.edu.cn and Yanglong Hou; hou@pku.edu.cn

**Supplementary Figures**


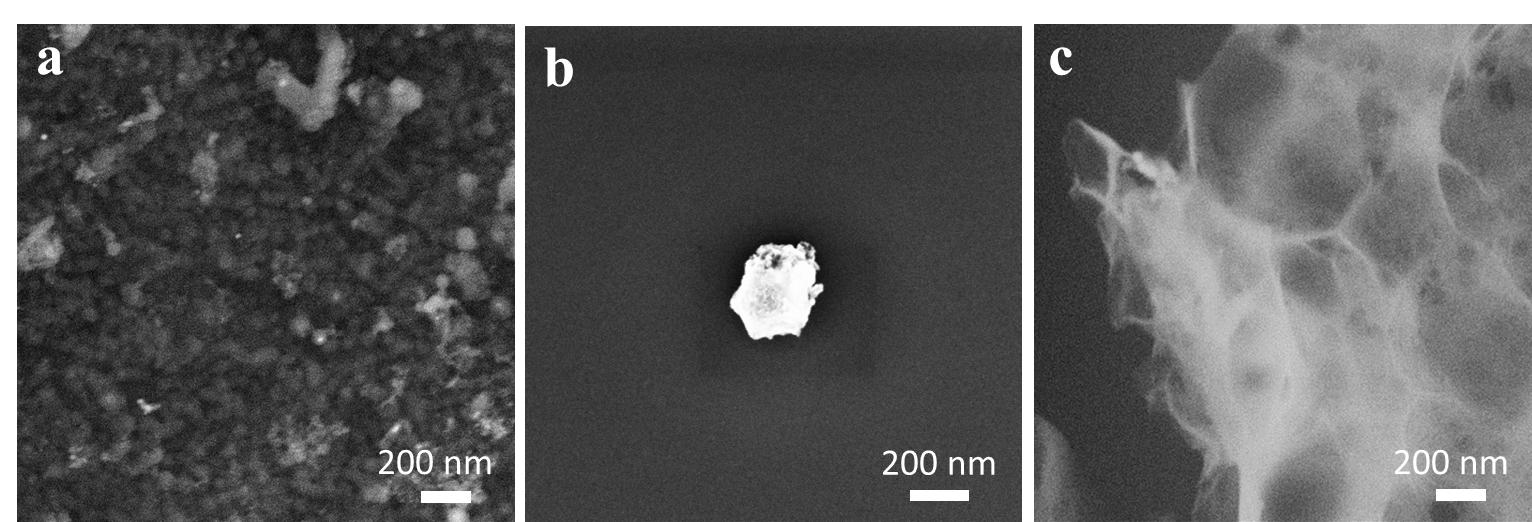


**Figure S1.** SEM images of (a) N/G, (b) Ni-N/C, and (c) GNs.


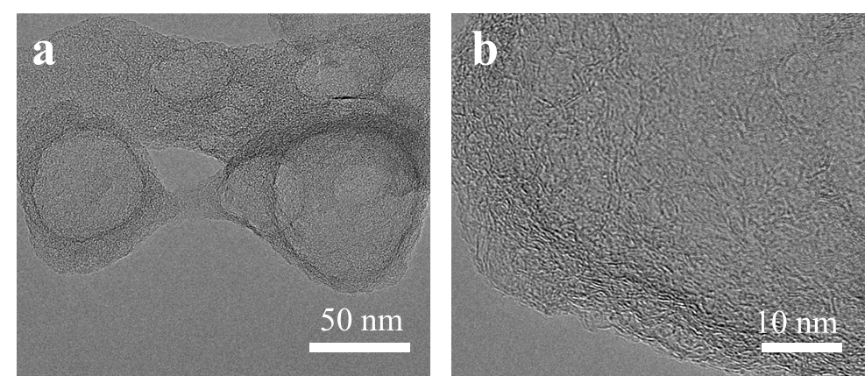


**Figure S2.** (a) TEM and (b) high resolution TEM (HRTEM) of Ni-N/G composite.


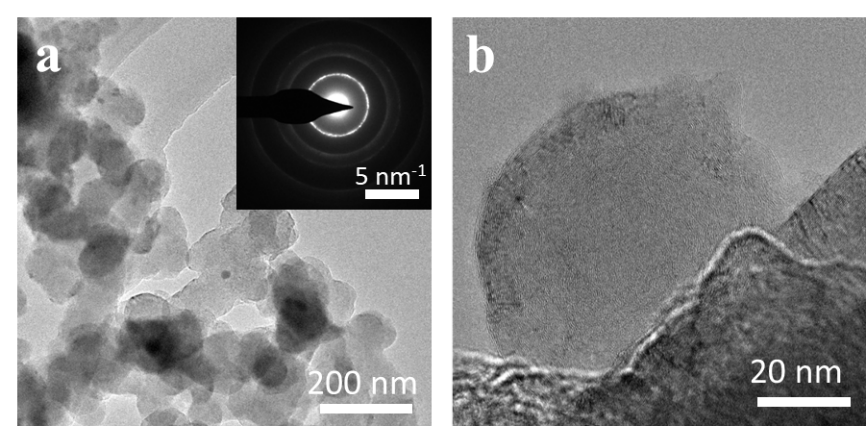


**Figure S3.** Representative electron microscopy images. (a) TEM (inset, corresponding SAEDP), and (b) HRTEM images of N/G sample.


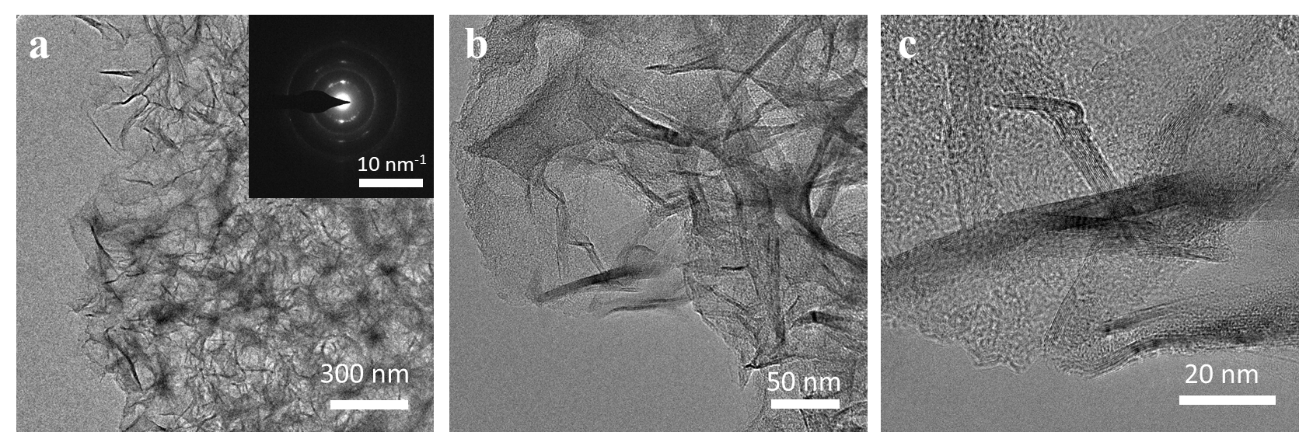


**Figure S4.** Representative electron microscopy images. (a-b) TEM (inset a, corresponding SAEDP), and (c) HRTEM images of GNs sample.

**
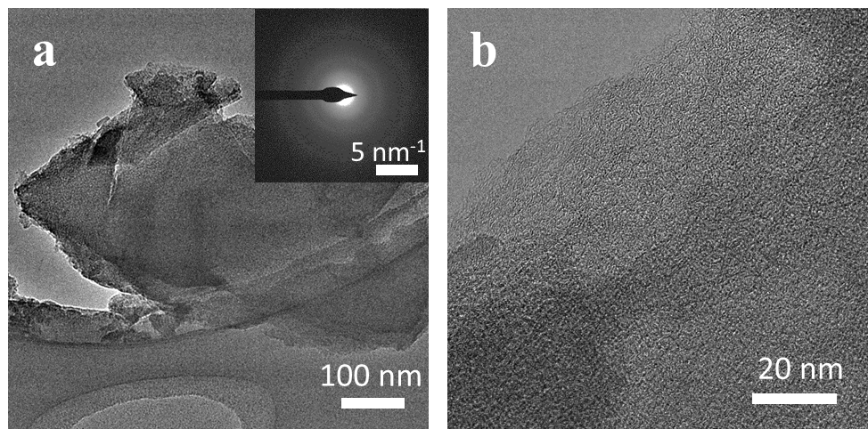
**

**Figure S5.** Representative electron microscopy images. (a) TEM (inset, corresponding SAEDP), and (b) HRTEM images of Ni-N/C sample.


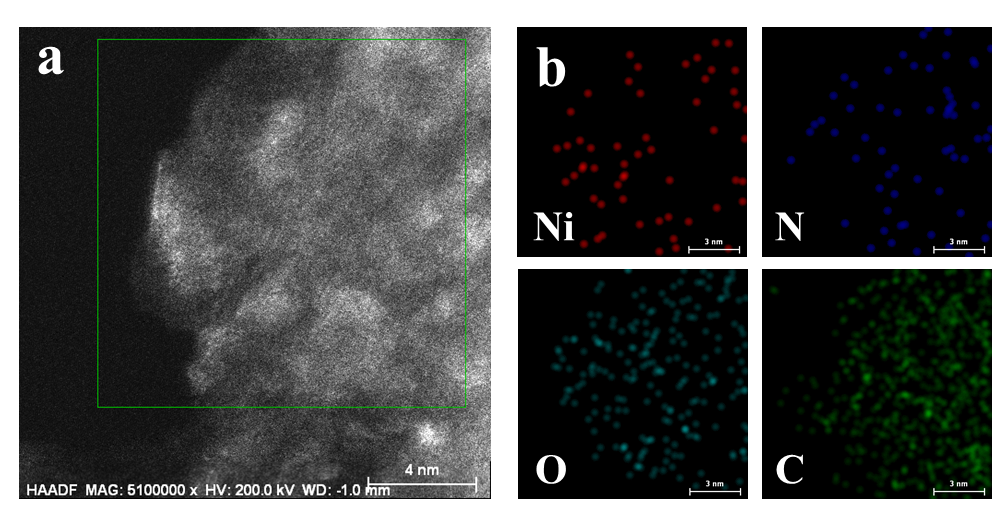


**Figure S6.** (a) HAADF-STEM image of the Ni-N/G sample and (b) corresponding EDX maps (colours) of individual element.

**
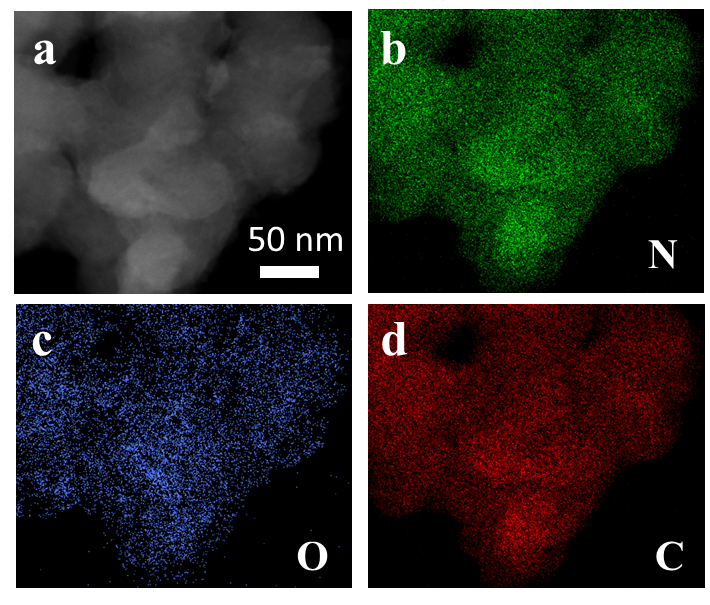
**

**Figure S7.** (a) STEM image of the N/G sample and (b-d) corresponding EDX maps (colours) of individual element.

**
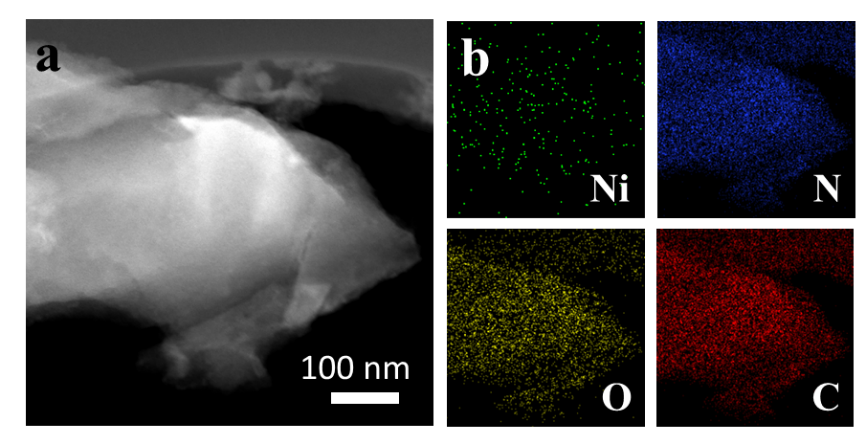
**

**Figure S8.** (a) STEM image of the Ni-N/C sample and (b) corresponding EDX maps (colours) of individual element.


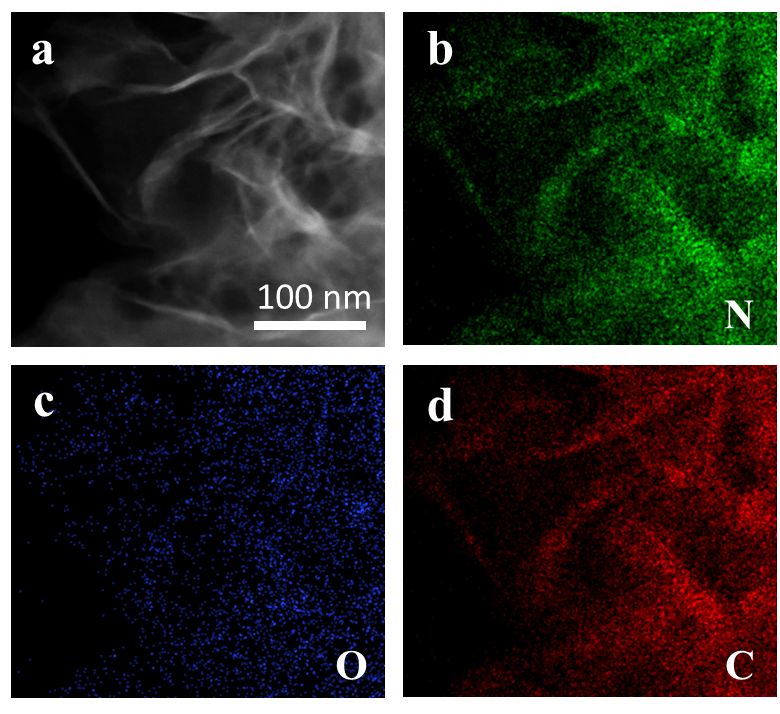


**Figure S9.** (a) STEM image of the GNs sample and (b-d) corresponding EDX maps (colours) of individual element.

**Figure S10.** XRD patterns of Ni-N/G, N/G, Ni-N/C and GNs.

**Figure S11.** Pore size distribution curves of Ni-N/G, N/G, Ni-N/C and GNs.

**Figure S12.** TGA curve of S@Ni-N/G, S@N/G, S@Ni-N/C and S@GNs in N_2_ atmosphere heated from room temperature to 600 °C with the ramping rate of 10 °C min^-1^.

**
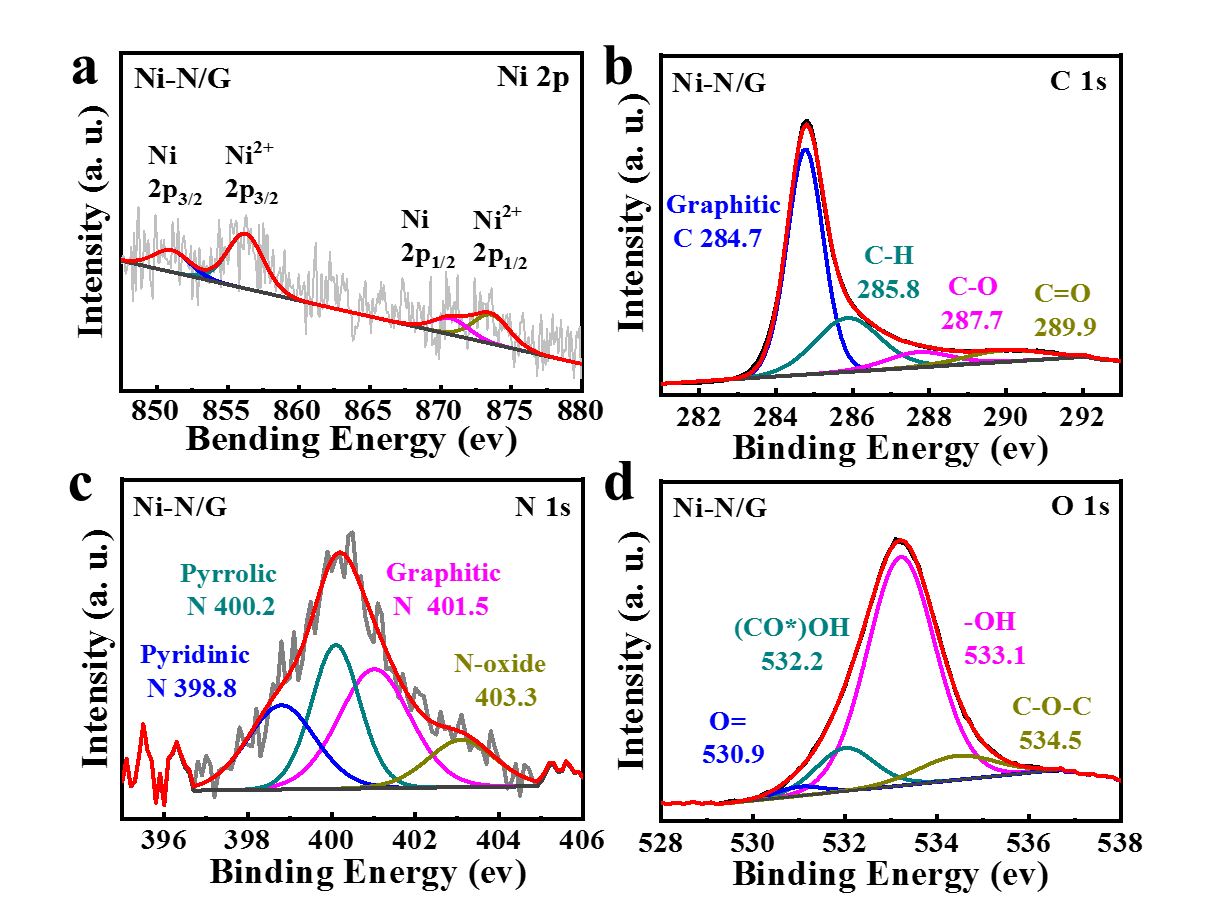
**

**Figure S13.** XPS high-resolution spectra of (a) Ni 2p, (b) C 1s, (c) N 1s, and (d) O 1s in Ni-N/G.

**
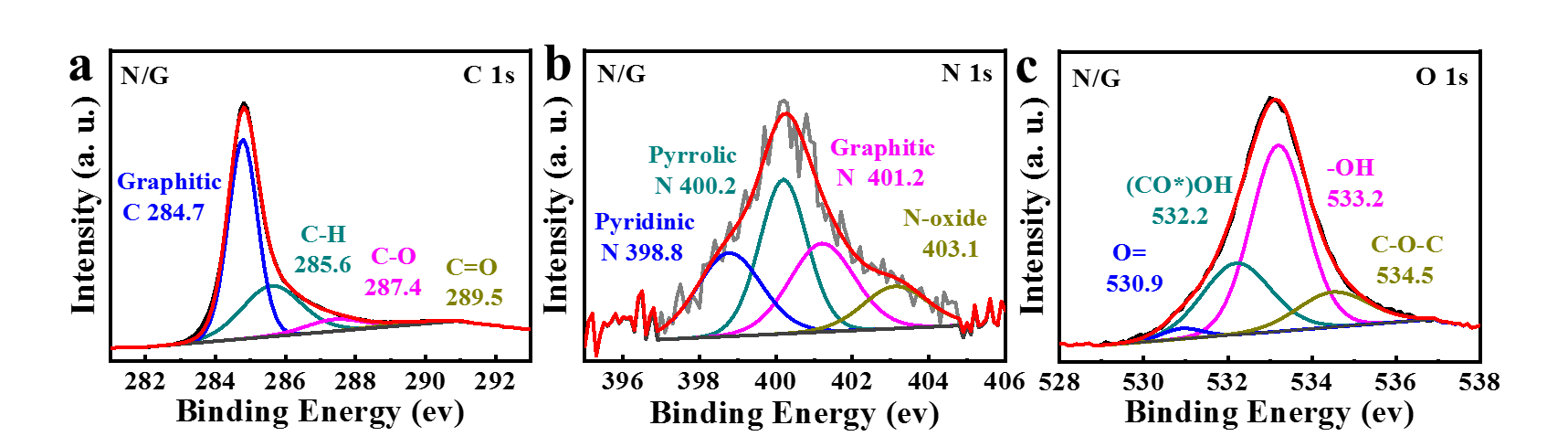
**

**Figure S14.** XPS high-resolution spectra of (a) C 1s, (b) N 1s, and (c) O 1s in N/G.

**
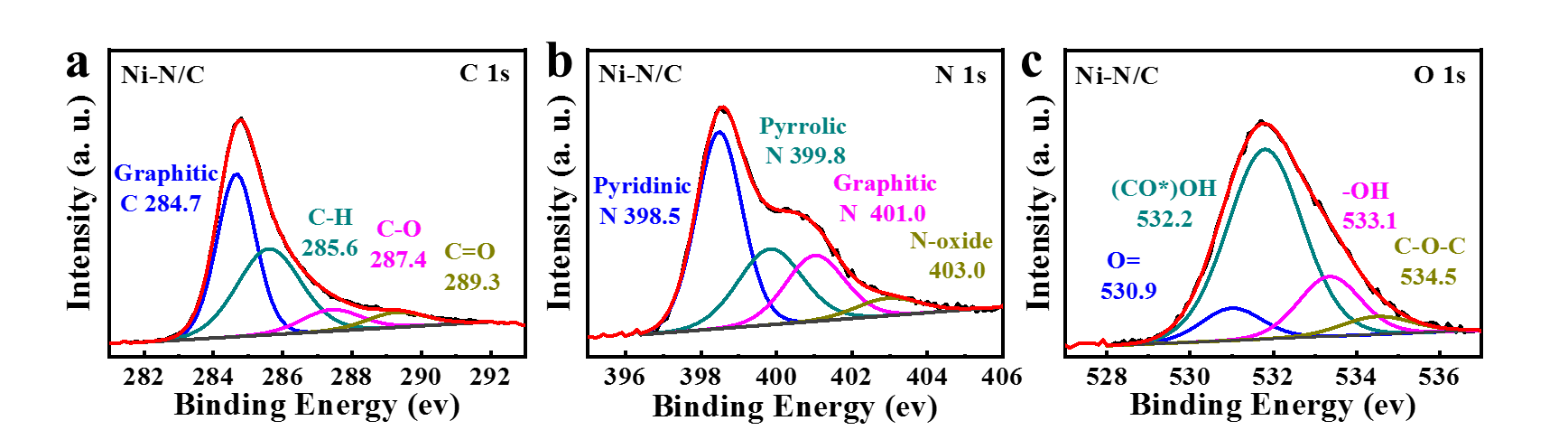
**

**Figure S15.** XPS high-resolution spectra of (a) C 1s, (b) N 1s, and (c) O 1s in Ni-N/C.

**
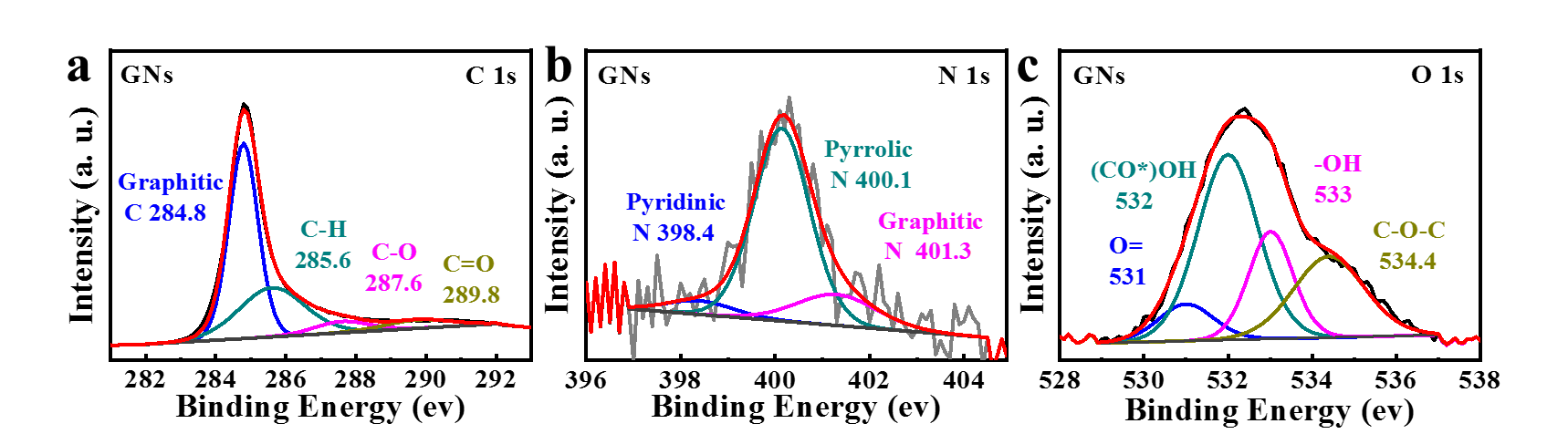
**

**Figure S16.** XPS high-resolution spectra of (a) C 1s, (b) N 1s, and (c) O 1s in GNs.


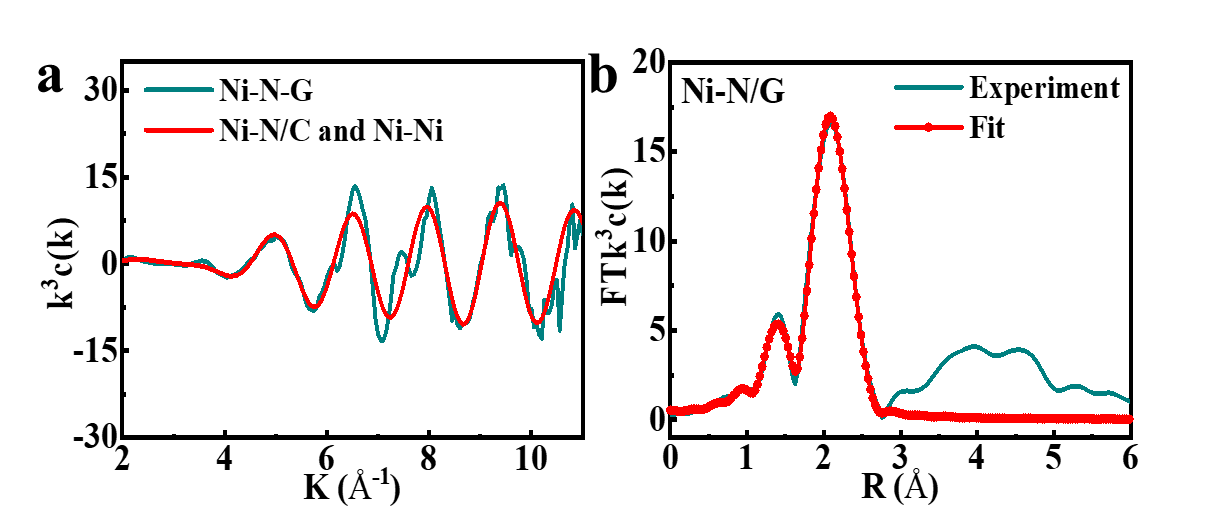


**Figure S17.** The EXAFS fitting in k space (a) and R-space (b) about the measured (green line) and fitting curves (red line) for Ni-N/G.


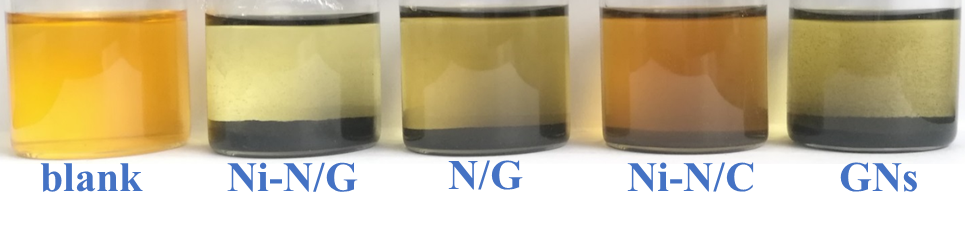


**Figure S18.** Experimental testing the polysulfide adsorption of Ni-N/G, N/G, Ni-N/C and GNs with Li_2_S_6_ as a representative lithium polysulfide.


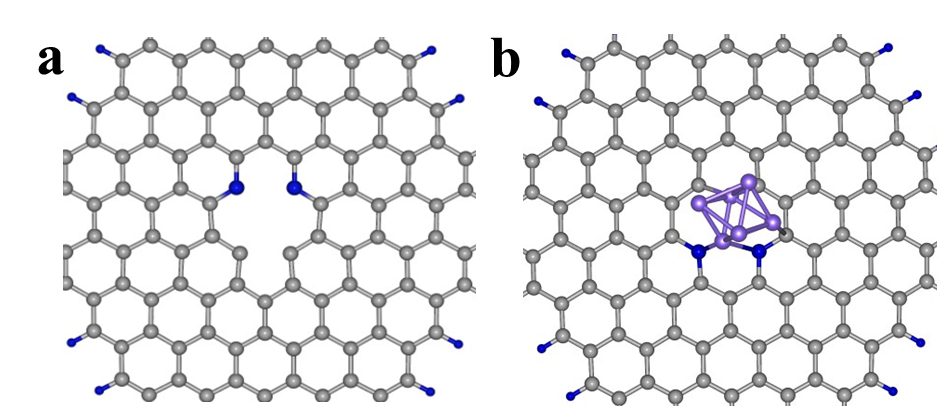


**Figure S19.** Structure of N/G (a) and Ni-N/G (b) used in first-principles calculations.


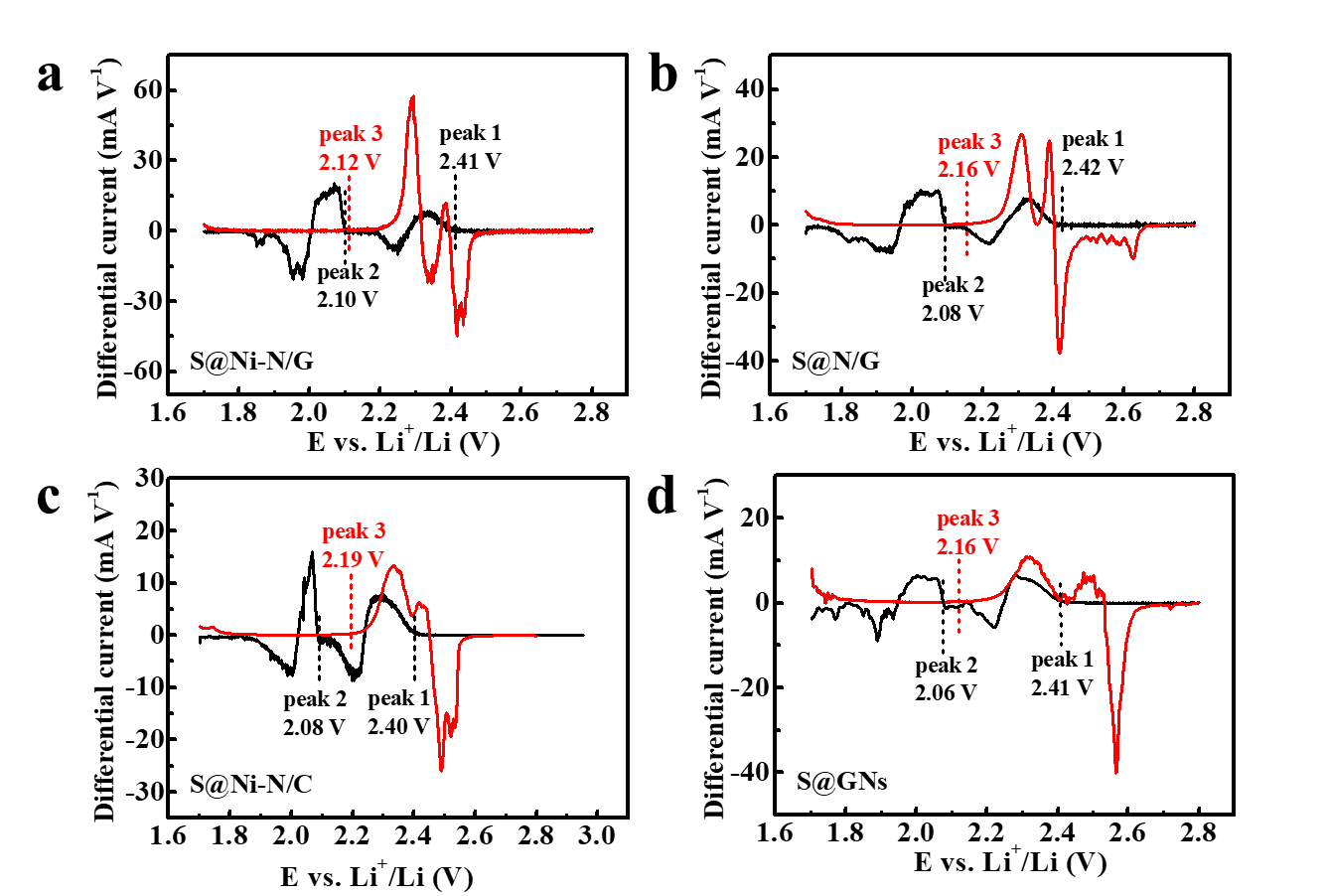


**Figure S20.** Differential CV curves (a-d) of S@Ni−N/G, S@N/G, S@Ni−N/C, and S@GNs cathodes verified from the CV profiles in **Figure 3B**. The corresponding onset potentials of redox peaks are attained at the current density of 10 μA cm^-2^. The baseline potentials and baseline current densities are defined as the values before the redox peaks, where the variation on current density is the smallest, namely dI/dV = 0.


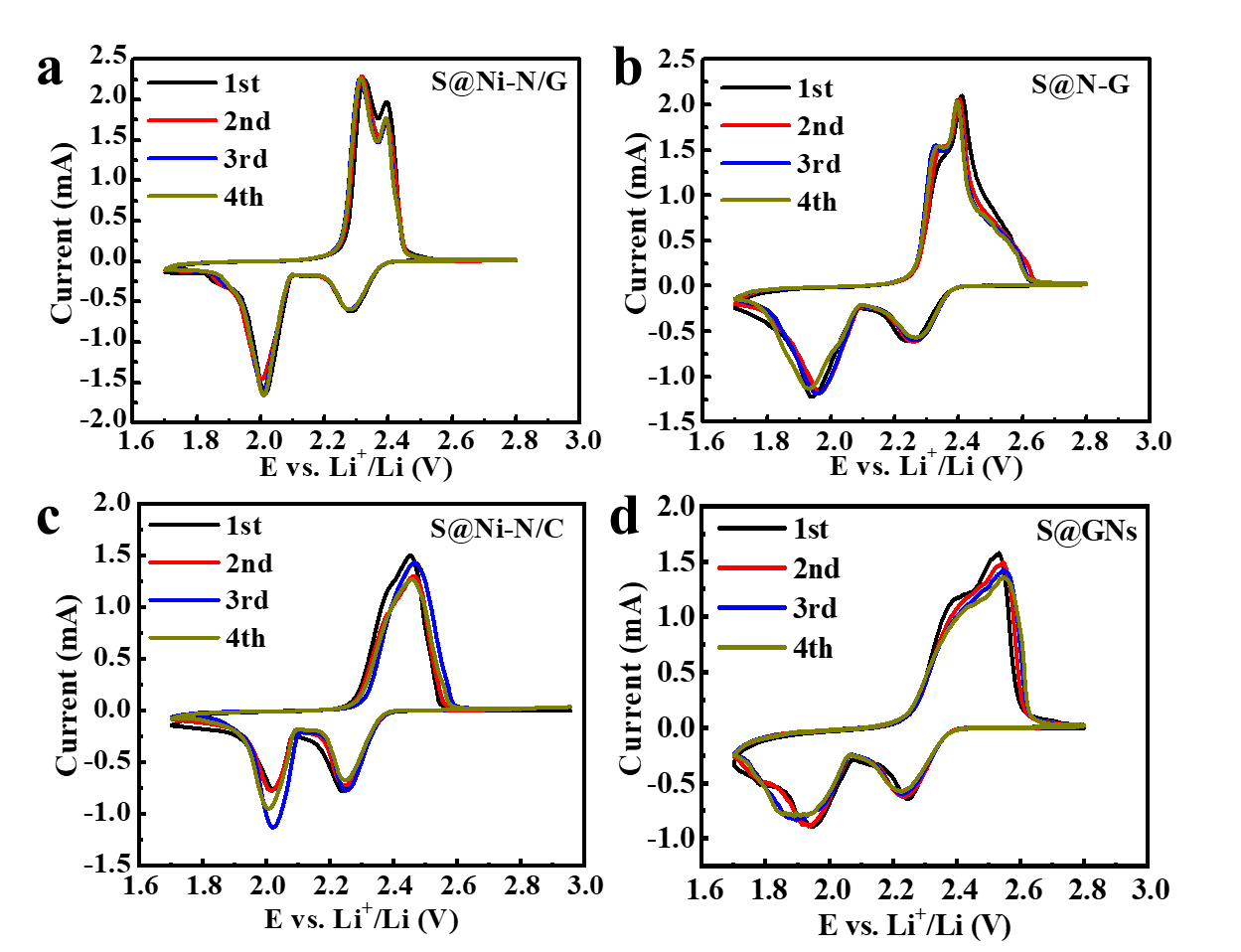


**Figure S21.** Electrochemical performances (a-d) of S@Ni−N/G, S@N/G, S@Ni−N/C, and S@GNs cathodes in Li−S batteries with the sulfur loading of 1.0 mg cm^−2^. CV curves over a voltage range of 1.7-2.8 V with sweep rate of 0.2 mV s^−1^.

**Figure S22.** Typical discharge-charge profiles of S@Ni−N/G composite at different current densities.

**Figure S23.** Electrochemical performances of S@Ni-N/G, S@N/G, S@Ni-N/C, and S@GNs cathodes in Li-S batteries with the sulfur loading of 1.0 mg cm^-2^. Long-term cycling performances and corresponding Coulombic efficiencies under 1.0 C.


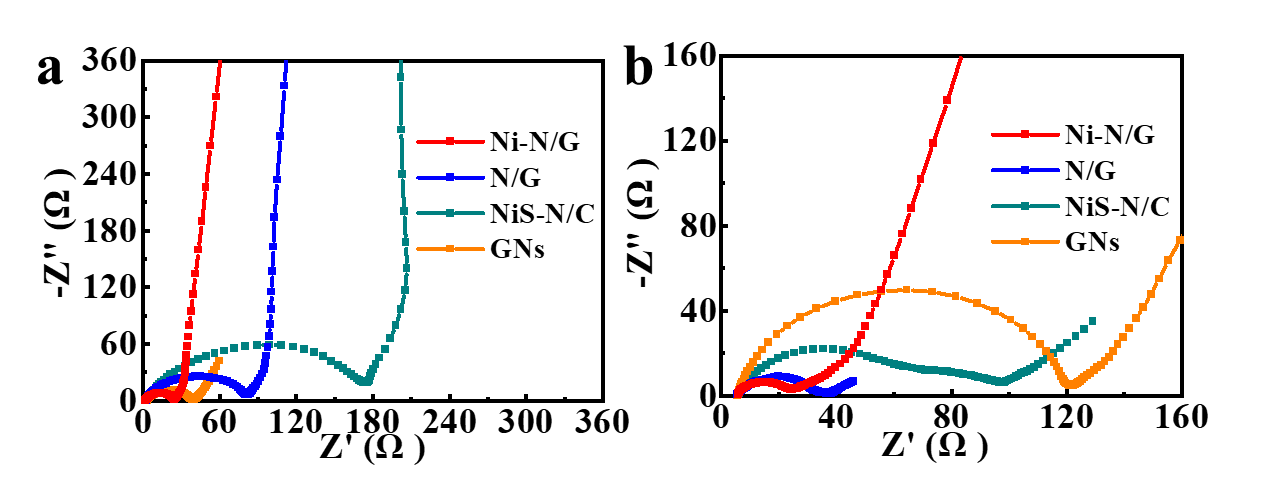


**Figure S24.** EIS spectra of S@Ni−N/G, S@N/G, S@Ni−N/C, and S@GNs cathodes (a) before and (b) after long-term cycling, revealing the much smaller charge transfer resistance of S@Ni−N/G cathode.

**Figure S25.** Cycling performance of Ni−N/G as the cathode material for LIBs within a voltage window of 1.7-2.8 V vs. Li/Li^+^. The ultra-low specific capacities indicate that the lithium storage capacity of pristine Ni−N/G has no contribution to the total discharge capacity of Li-S batteries.

**Figure S26.** Cycling performance and Coulombic efficiencies of S@Ni-N/G with different areal loading of sulfur at 0.1 C.


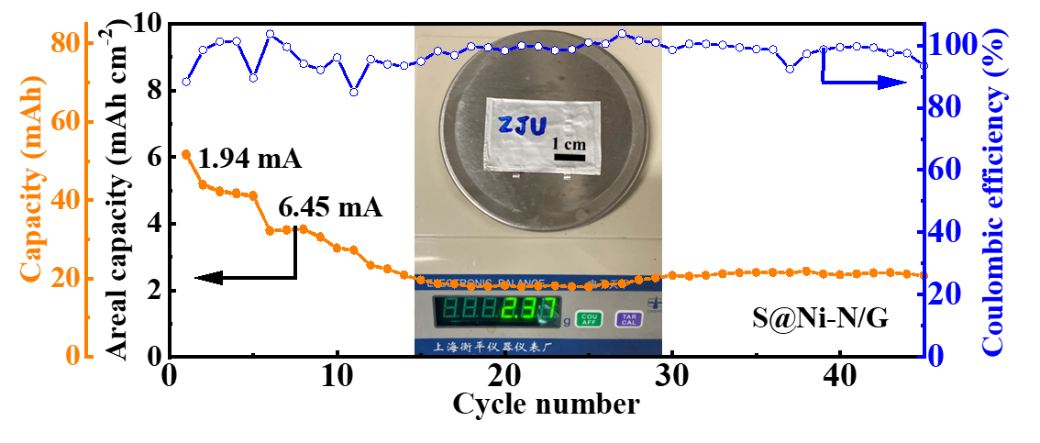


**Figure S27.** Cycling performance and Coulombic efficiencies of S@Ni−N/G based soft-packed Li−S battery. Insert: the photograph of the soft pack Li−S battery assembled with the S@Ni−N/G cathode and lithium foil anode.

**Supporting Tables**

**Table S1.** Atomic percentage ratios of different elements in Ni-N/G according to the EDX results of HADDF-STEM.

| Element | series | Net | [wt.%] | [norm. wt.%] | [norm. at.%] | Error in wt.% (3 Sigma) |
| --- | --- | --- | --- | --- | --- | --- |
| Ni | L-series | 44 | 0.70 | 0.70 | 0.15 | 0.388 |
| C | K-series | 644 | 94.88 | 94.88 | 96.32 | 4.157 |
| N | K-series | 42 | 1.42 | 1.42 | 1.24 | 0.744 |
| O | K-series | 198 | 3.00 | 3.00 | 2.29 | 0.769 |

**Table S2.** BET surface area, pore volume and pore diameter of Ni−N/G, N/G, Ni-N/C and GNs.

| Sample | S_BET_ (m^2^ g^-1^) | Pore volume (cm^3^ g^-1^) | Pore diameter (nm) |
| --- | --- | --- | --- |
| Ni-N/G | 919.9 | 0.948 | 3.86 |
| N/G | 724.0 | 0.735 | 3.89 |
| Ni-N/C | 160.7 | 0.097 | 2.42 |
| GNs | 742.9 | 0.79 | 3.61 |

**Table S3.** Atomic percentage ratios of different elements in Ni-N/G and control samples according to the results of XPS.

| Sample | Ni [at.%] | C [at.%] | N [at.%] | O [at.%] |
| --- | --- | --- | --- | --- |
| Ni-N/G | 0.16 | 94 | 1.35 | 4.49 |
| N/G | - | 94.24 | 1.22 | 4.54 |
| Ni-N/C | 0.24 | 86.13 | 6.4 | 7.23 |
| GNs | - | 95.37 | 0.56 | 4.07 |

**Table S4.** Atomic percentage ratios of different N types in Ni-N/G and control samples according to the results of XPS.

| Sample | pyridinic N [at.%] | pyrrolic N [at.%] | graphitic N [at.%] | N-oxide [at.%] |
| --- | --- | --- | --- | --- |
| Ni-N/G | 23.79 | 40.81 | 26.18 | 9.22 |
| N/G | 25.46 | 34.58 | 27.05 | 12.91 |
| Ni-N/C | 47.84 | 25.68 | 20.54 | 5.94 |
| GNs | 6.58 | 75.86 | 17.56 | - |

**Table S5.** EXAFS parameters for Ni-N/G, Ni foil, NiO and Ni porphyrin.

| Sample | Ni-Ni | | Ni-N | | Ni-C | | χ*_ν_*^2^ | R-factor |
| --- | --- | --- | --- | --- | --- | --- | --- | --- |
|  | R (Å) | CN | R (Å) | CN | R (Å) | CN |  |  |
| Ni-N/G | 2.45 ± 0.05 | 5.70 ± 0.41 | 1.81 ± 0.05 | 0.33 ± 0.05 | 2.85 ± 0.10 | 0.54 ± 0.05 | 78 | 0.0113 |
| Ni foil | - | - | 2.49 | 12 | - | - | - | - |
| NiO | 2.95 | 12 | 2.09 (O) | 6 | - | - | - | - |
| Ni porphyrin | - | - | 1.87 | 4 | - | - | - | - |

**Table S6.** The binding energies between different lithium polysulfides and substrates, based on Ni-N/G compared with other currently reported sulfur host materials in the literatures. Energy unit: electronvolts. Zero-point corrected.

| Ref. | Substrate | S_8_ | Li_2_S_8_ | Li_2_S_6_ | Li_2_S_4_ | Li_2_S_2_ | Li_2_S |
| --- | --- | --- | --- | --- | --- | --- | --- |
| This work | Ni-N/G | -0.57 | -3.64 | -3.51 | -3.28 | -2.90 | -2.75 |
|  | N/G | -0.28 | -1.28 | -1.47 | -1.94 | -2.58 | -1.45 |
| 11 | CoS | -1.99 | -3.54 | -3.11 | -2.79 | -3.24 | -3.82 |
| 12 | MMNC | -0.51 | -0.74 | -0.67 | -0.70 | -0.85 | -0.85 |
| 13 | VN-NBs | -2.21 | -3.46 | -3.01 | -3.27 | -3.14 | -3.86 |
|  | V_2_O_5_-NBs | -1.43 | -1.92 | -1.61 | -1.95 | -2.46 | -2.64 |
| 14 | α-Fe_2_O_3_ | -2.04 | -3.78 | -4.11 | -4.09 | -4.20 | -4.85 |
|  | Graphene | -0.89 | -1.10 | -0.93 | -0.72 | -0.74 | -0.65 |
| 15 | V_2_O_5_ | ~ -0.80 | ~ -3.65 | ~ -3.70 | -3.73 | ~ -3.90 | ~ -4.20 |
|  | MoO_3_ | -0.75 | ~ -2.00 | ~ -2.20 | -2.85 | ~ -2.95 | ~ -3.77 |
|  | TiS_2_ | ~ -0.80 | ~ -1.15 | ~ -0.90 | -1.40 | ~ -1.73 | ~ -3.08 |
|  | ZrS_2_ | ~ -0.80 | ~ -1.20 | ~ -0.95 | ~ -1.45 | ~ -1.75 | ~ -3.10 |
|  | VS_2_ | -0.85 | ~ -1.23 | ~ -1.00 | ~ -1.55 | ~ -1.80 | ~ -3.20 |
|  | NbS_2_ | ~ -0.80 | ~ -1.40 | ~ -1.30 | -1.80 | ~ -2.05 | ~ -3.40 |
|  | MoS_2_ | ~ -0.80 | ~ -0.80 | ~ -0.75 | -0.77 | ~ -1.00 | ~ -0.95 |
|  | TiCl_2_ | ~ -0.80 | ~ -0.75 | ~ -0.60 | -0.38 | ~ -0.72 | ~ -0.71 |
|  | ZrCl_2_ | ~ -0.80 | ~ -0.75 | ~ -0.60 | ~ -0.38 | ~ -0.72 | ~ -0.71 |

**Table S7.** Summary of electrochemical impedance spectroscopy of the initial Ni-N/G, N/G, Ni-N/C and GNs samples at open circuit voltage.

| 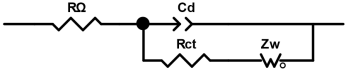 | | | | Constant phase Angle elements | | | |
| --- | --- | --- | --- | --- | --- | --- | --- |
| Catalyst | solution resistance (Ω) | charge transfer resistance (Ω) | Warburg resistance (Ω) | Cd-T | Cd-P | Zw-T | Zw-P |
| Ni-N/G | 2.79 | 22.92 | 17.8 | 3.23E-5 | 0.795 | 0.211 | 0.460 |
| N/G | 2.65 | 79.98 | 49.42 | 2.08E-5 | 0.751 | 0.313 | 0.475 |
| Ni-N/C | 2.09 | 180.4 | 95.39 | 2.21E-5 | 0.734 | 0.165 | 0.479 |
| GNs | 3.01 | 35.49 | 13.18 | 2.13E-5 | 0.758 | 2.885 | 0.365 |

**Table S8.** Summary of electrochemical impedance spectroscopy of the Ni-N/G, N/G, Ni-N/C and GNs samples at open circuit voltage after 400 cycles of 1C.

| 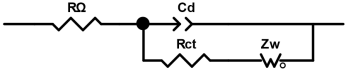 | | | | Constant phase Angle elements | | | |
| --- | --- | --- | --- | --- | --- | --- | --- |
| Catalyst | solution resistance (Ω) | charge transfer resistance (Ω) | Warburg resistance (Ω) | Cd-T | Cd-P | Zw-T | Zw-P |
| Ni-N/G | 5.59 | 18.18 | 40.89 | 1.24E-5 | 0.800 | 0.736 | 0.419 |
| N/G | 5.54 | 30.64 | 63.84 | 3.12E-5 | 0.662 | 359.4 | 0.414 |
| Ni-N/C | 5.65 | 82.58 | 216.1 | 4.92E-5 | 0.662 | 384.3 | 0.466 |
| GNs | 3.18 | 114.5 | 75.47 | 2.42E-6 | 0.915 | 1.708 | 0.420 |

**Table S9.** Electrochemical performances of sulfur cathodes based on Ni-N/G compared with other currently reported sulfur host materials in the literatures.

| Ref. | Sulfur hosts (morphology) | Sulfur weight (wt.%) | Sulfur loading in electrodes (mg cm^-2^) | Current rate (C) | Cycle number | Discharge capacity (mAh g^-1^) | Discharge areal capacity (μAh cm^-2^) | Capacity retention (%) |
| --- | --- | --- | --- | --- | --- | --- | --- | --- |
| This work | Ni-N/G | 74.1 | 2.3 | 0.1 | 150 | 734 | 1688 | 76.1 |
|  |  |  | 2.0 | 0.2 | 100 | 954 | 1908 | 86.4 |
|  |  |  | 1.0 | 1 | 400 | 538 | 538 | 62.9 |
| 36 | Ti_4_O_7_ particles | ~50 | ~1.1 | 0.1 | 300 | 871 | ~958 | 71.6 |
| 34 | Fe-PNC/S | ~70 | 1.3 | 0.1 | 300 | 427 | 555.1 | 40 |
| 37 | Mesoporous Magnéli Ti_4_O_7_ microspheres | 55.0 | ~0.5 | 0.2 | 400 | 1000 | ~500 | 84.5 |
| 38 | Carbon/TiN | - | 1.2 | 0.2 | 50 | ~800 | ~960 | ~69 |
| 39 | V_2_O_5_ | ~65 | 1.5 | 0.2 | 450 | 592 | 888 | 74.0 |
| 40 | cloth/Al_2_O_3_ layer | 59 | 3.74 | 0.2 | 40 | 766 | 2865 | ~70 |
| 49 | Ti_3_C_2_T_x_ (4 h)-GN | 75 | 7.3 | 0.2 | 200 | ~510 | ~3723 | 57.7 |
| 11 | CNTs/CoS-NSs | 76.5 | ~1.0 | 0.2 | 100 | 1154 | ~1154 | 86.2 |
|  |  |  |  | 1.0 | 1000 | 675 | ~675 | 68.0 |
| 12 | CeO_2_/MMNC nanospheres | 63.6 | 1.4 | 0.2 | 200 | 1066 | 1492 | 78.0 |
|  |  |  |  | 1.0 | 500 | 836 | 1170 | 61.8 |
| 9 | Co-N/G | 90 | 2.0 | 0.2 | 100 | ~1000 | ~2000 | ~80 |
|  |  |  |  | 1.0 | 500 | 681 | 1362 | 73.5 |
| 46 | TiO_2_ hollow nanospheres | ~61 | ~0.5 | 1.0 | 1000 | 690 | ~345 | 67.0 |
| 44 | PPy-MnO_2_ | 70.0 | 1.0 | 1.0 | 500 | 550 | 550 | 65.0 |
| 13 | VN nanobubbles | 78.2 | 1.2 | 1.0 | 1000 | 837 | 1004 | 76.1 |
| 41 | TiO_2_-TiN | 88.0 | 1.2 | 1.0 | 1000 | ~750 | 900 | - |
| 35 | NC:SAFe | 51.6 | 1.4 | 1.0 | 200 | 790 | 1106 | 75 |
| 43 | Co_4_N nanoflowers | 59.5 | ~1.5 | 1.0 | 100 | 1000 | ~1500 | - |
| 45 | CNTs/V_2_O_5_ layer | ~66.6 | 2.0 | 1.0 | 250 | 939 | 1878 | 87.9 |
| 48 | Co@C_3_N_4_ | - | 2 | 1.0 | 200 | ~980 | ~1960 | ~81.7 |
| 42 | Hollow Co_3_S_4_ polyhedra | ~50 | 2.5 | 1.0 | 450 | 610 | 1525 | 64.5 |
